# Revision of the Genus *Rhagastis* Rothschild & Jordan, 1903 (Lepidoptera: Sphingidae) from China, Based on Morphological and Phylogenetic Analyses

**DOI:** 10.3390/insects15050359

**Published:** 2024-05-15

**Authors:** Zhuo-Heng Jiang, Jia-Xin Wang, Zhen-Bang Xu, Ian J. Kitching, Chia-Lung Huang, Shao-Ji Hu, Yun-Li Xiao

**Affiliations:** 1 Yunnan Key Laboratory of International Rivers and Transboundary Eco-Security, Yunnan University, Kunming 650500, China; jzhsphingidae@163.com (Z.-H.J.); zhenbangxumm@gmail.com (Z.-B.X.); 2 Institute of International Rivers and Eco-Security, Yunnan University, Kunming 650500, China; 3 School of Life Science, Westlake University, Hangzhou 310023, China; 4 Hubei Key Laboratory of Economic Forest Germplasm lmprovement and Resources Comprehensive Utilization, Huanggang Normal University, Huanggang 438000, China; 13886408576@139.com; 5 College of Agriculture, Yangtze University, Jingzhou 434000, China; 6 Guangxi Institute of Botany, Chinses Academy of Sciences, Guilin 541006, China; 7 Natural History Museum, Cromwell Road, London SW7 5BD, UK; i.kitching@nhm.ac.uk; 8 Fujian Key Laboratory on Conservation and Sustainable Utilization of Marine Biodiversity, Fuzhou Institute of Oceanography, College of Geography and Oceanography, Minjiang University, Fuzhou 350108, China; 9066@mju.edu.cn

**Keywords:** hawkmoth, *Rhagastis*, DNA barcodes, molecular phylogeny, genitalic structure

## Abstract

**Simple Summary:**

Simple Summary: The genus *Rhagastis* Rothschild & Jordan, 1903 (Lepidoptera, Sphingidae, Macroglossinae, Macroglossini) currently comprises sixteen species, ten of which are found in China; however, complex and confusing taxonomic issues have existed for a long time. We performed an analysis based on a 658-bp region of the COI mitochondrial gene (DNA barcode) and morphological characteristics such as the wing pattern and genital structure of the *albomarginatus* group, the *castor* group, the *olivacea* group and other species, demonstrating that subspecies of *Rhagastis albomarginatus* and *R. castor* should be treated as good species, *i.e.*, *Rhagastis dichroae* stat. nov., *R. everetti* stat. nov., *R. aurifera* stat. rev., *R. chinensis* stat. nov., *R. formosana* stat. nov., and *R. jordani* stat. rev.

**Abstract:**

Here, the taxonomy of the genus *Rhagastis* Rothschild & Jordan, 1903 (Lepidoptera, Sphingidae, Macroglossinae, Macroglossini) from China is revised based on differences in wing morphology, male and female genitalia, and the phylogenetic relationship of the DNA barcodes. Subspecies of *Rhagastis albomarginatus* (Rothschild, 1894) and *R. castor* (Walker, 1856) are treated as “good” species, namely *Rhagastis dichroae* Mell, 1922 stat. nov.; *R. everetti* Rothschild & Jordan, 1903 stat. nov.; *R. aurifera* (Butler, 1875) stat. rev.; *R. chinensis* Mell, 1922 stat. nov.; *R. formosana* Clark, 1925 stat. nov.; and *R. jordani* Oberthür, 1904 stat. rev. The distribution maps, biological notes, and ecological records of the genus *Rhagastis* Rothschild & Jordan, 1903 from China are given, and a species inventory of genus *Rhagastis* in the world is also included.

## 1. Introduction

The genus *Rhagastis* Rothschild & Jordan, 1903 (Lepidoptera, Sphingidae, Macroglossinae, Macroglossini), with type species *Rhagastis velata* (Walker, 1866), distributed from the Eastern Palearctic to the Oriental regions and Madagascar, previously comprising sixteen species [1,2]. Ten of them are found in China, namely *R. acuta* (Walker, 1856); *R. albomarginatus* (Rothschild, 1894); *R. binoculata* Matsumura, 1909; *R. castor* (Walker, 1856); *R. confusa* Rothschild & Jordan, 1903; *R. gloriosa* (Butler, 1875); *R. lunata* (Rothschild, 1900); *R. mongoliana* (Butler, 1876); *R. olivacea* (Moore, 1872); and *R. velata* (Walker, 1866) [3,4,5,6,7,8,9,10]. The other six species not found in China are *R. castanea* (Moore, 1872); *R. diehli* Haxaire & Melichar, 2010; *R. lambertoni* Clark, 1923; *R. meridionalis* Gehlen, 1928; *R. rubetra* Rothschild & Jordan, 1907; and *R. trilineata* Matsumura, 1921 [10,11,12,13,14,15].

**Figure 1:**
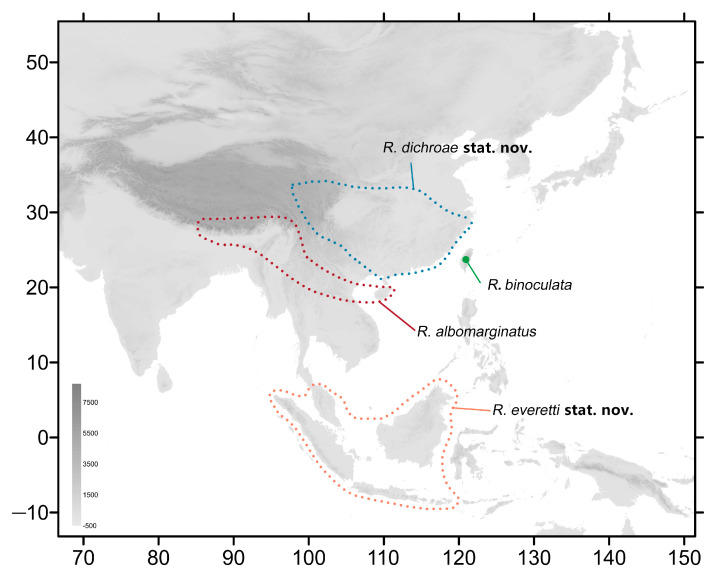
Distribution of species of the *albomarginatus* group of genus *Rhagastis* in this study. The blue dotted line indicates the range of *R. dichroae* stat. nov., the red dotted line indicates the range of *R. albomarginatus*, the orange dotted line indicates the range of *R. everetti* stat. nov., and the green circle indicates the range of *R. binoculata*.

**Figure 2:**
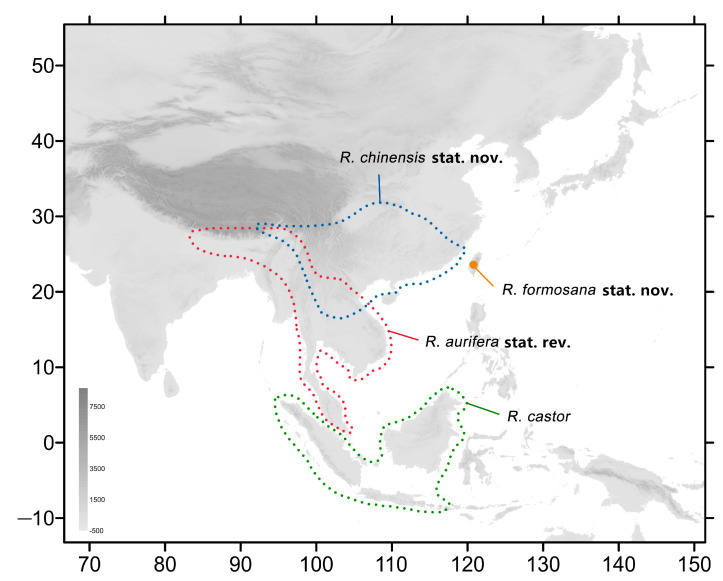
Distribution of species of the *castor* group of genus *Rhagastis* in this study. The blue dotted line indicates the range of *R. chinensis* stat. nov., the red dotted line indicates the range of *R. aurifera* stat. rev., the green dotted line indicates the range of *R. castor*, and the orange circle indicates the range of *R. formosana* stat. nov.

The species currently known as *Rhagastis albomarginatus* (Rothschild, 1894) is a wide-ranged moth containing a few subspecies, namely the nominate subspecies found in southwestern China, Nepal, Bhutan, India, Indochina, and parts of Malaysia and Indonesia; ssp. *dichroae*, found in eastern, central, and southeastern China; and ssp. *everetti*, found in parts of Malaysia and Indonesia [3,16] (Figure 1). Another species, *R. castor* (Walker, 1856), also contains a number of subspecies, some of which were originally described as species. *R. castor* currently consists of four subspecies: the nominate subspecies found in parts of Malaysia and Indonesia; ssp. *aurifera* in southwestern China, Nepal, Bhutan, India, and Indochina; ssp. *jordani* found in central and southwestern China; and ssp. *formosana*, which is endemic to Taiwan Island of China [7,16,17,18] (Figure 2).

**Figure 3:**
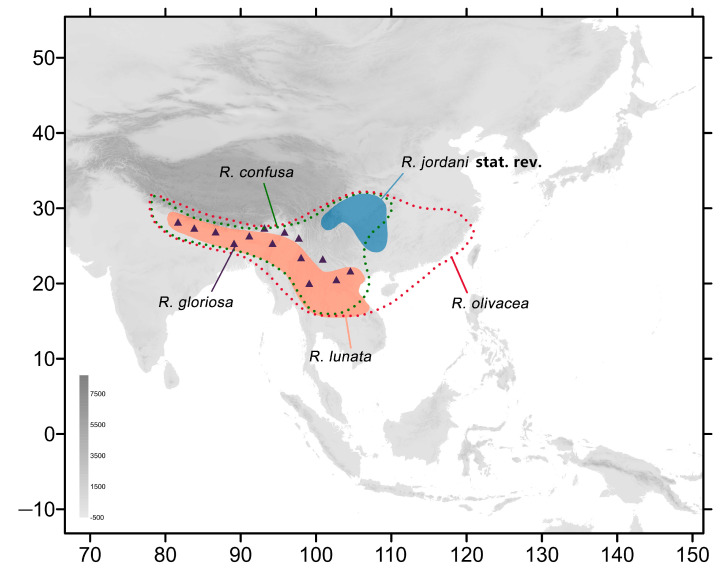
Distribution of species of the *olivacea* group of genus *Rhagastis* in this study. The green dotted line indicates the range of *R. confusa*, the red dotted line indicates the range of *R. olivacea*, the blue patch indicates the range of *R. jordani* stat. rev., the orange patch indicates the range of *R. lunata*, and purple triangles indicate the range of *R. gloriosa*.

**Figure 4:**
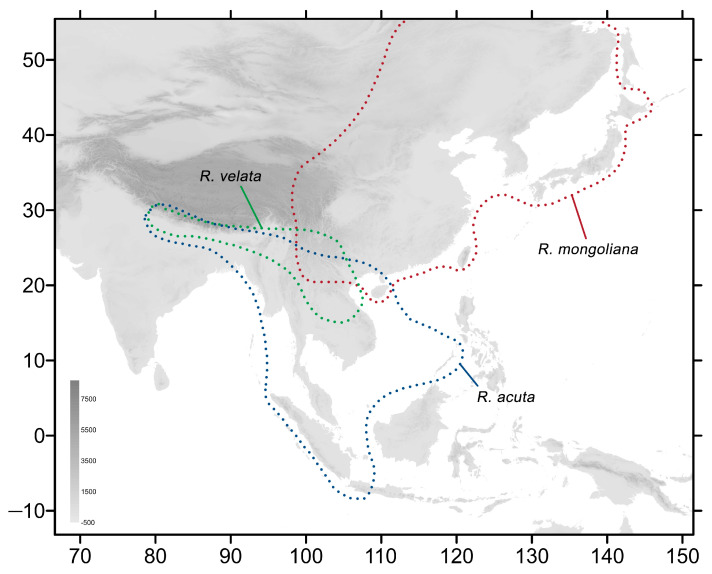
Distribution of the other species of genus *Rhagastis* in this study. The blue dotted line indicates the range of *R. acuta*, the red dotted line indicates the range of *R. mongoliana*, and the green dotted line indicates the range of *R. velata*.

To revise this complex genus and to clarify the taxonomic confusion between species and subspecies, between 2015 and 2023, we collected various *Rhagastis* specimens from different localities to perform the analyses in the present study using morphological and phylogenetic approaches. After comparing the differences in wing patterns and genitalia, as well as the differences in DNA barcodes, we designate *Rhagastis dichroae* Mell, 1922 stat. nov.; *R. everetti* Rothschild & Jordan, 1903 stat. nov.; *R. aurifera* (Butler, 1875) stat. rev.; and *R. formosana* Clark, 1925 stat. nov. as good species. We also elevate the following to good species: *R. chinensis* Mell, 1922 stat. nov. from *R. aurifera chinensis* Mell, 1922, previously a synonym of *R. castor jordani*, and *R. jordani* Oberthür, 1904 stat. rev., which is more closely related to *R. olivacea* than to *R. castor* and is found only in central and southwestern China (Figure 3). Other *Rhagastis* species from China (Appendix A) are also listed (Figure 4), illustrated, and discussed in detail in this article with regard to their distribution ranges and their biological and ecological notes.

## 2. Materials and Methods

### 2.1. Taxon Sampling

The specimens of genus *Rhagastis* used in this study were sampled for both morphological and molecular analyses. Most specimens were collected and dried at room temperature in paper triangles and stored at −20 °C until use. Some samples were directly spread after collection to avoid abrasion of the scales of the head, thorax, and abdomen.

For each individual used in the molecular analysis, two legs from the same side were taken for DNA extraction before the specimens were rehydrated for spreading. Some se-quences of genus *Rhagastis* were downloaded from the Barcode of Life Database v.4 (BOLD) (http://www.boldsystems.org, accessed on 13 January 2024) as supplementary samples for phylogenetic analysis. In addition, sixteen sequences, including one individual of *R. castor aurifera*, two individuals of *R. binoculata*, two individuals of *R. confusa*, one individual of *R. castor formosana*, two individuals of *R. gloriosa*, three individuals of *R. lunata*, three individuals of *R. olivacea*, and two individuals of *R. velata* were also downloaded from BOLD for this study. An individual of *Phyllosphingia dissimilis* (OQ579169) and an individual of *Meganoton analis* (OQ589939) were chosen as outgroups. The collecting data, BOLD sample IDs, and GenBank accession numbers are listed in Table 1.

### 2.2. DNA Extraction and Amplification

The phenol–chloroform protocol was used to extract genomic DNA. The legs were-homogenized in protease buffer containing 450 µL STE (10 mmol/L Tris-HCl, 1 mmol/L EDTA, 100 mmol/L NaCl, pH = 8.0), 25 µL Proteinase K (20 mg/mL), and 75 µL SDS (10%) and incubated at 55 °C for 12 h to rehydrate and lyse the tissue. The subsequent extraction protocol followed the method of Xu et al. [19], and the resultant genomic DNA was preserved at −40 °C.

DNA amplification followed the method of Xu et al. [19]. The polymerase chain reaction (PCR) was carried out in a 25 µL system using the TaKaRa Ex *Taq* Kit (TaKaRa Biotechnology Co., Ltd., Dalian, China). The system contained 2.5 µL 10× PCR buffer, 2.0 µL MgCl_2_ (2.5 mmol/L), and 2.0 µL dNTP mixture (2.5 mmol/L each). The mitochondrial *cox1* gene fragment (the DNA barcode) was amplified and sequenced with the primers LCO1490 (5′-GGT CAA ATC ATA AAG ATA TTG-3′) and HCO2198 (5′-TAA ACT TCA GGG TGA CCA AAA AAT CA-3′) [20]. The PCR thermal profile consisted of an initial denaturation at 95 °C for 3 min; 30 cycles of denaturation at 94 °C for 1 min, annealing at 50 °C for 1 min, and elongation at 72 °C for 1 min; then a final elongation at 72 °C for 5 min. Sequencing was undertaken using an ABI Prism 3730 sequencer (Applied Biosystems, Foster City, CA, USA).

### 2.3. Genetic Distances and Phylogenetic Reconstruction

We proofread and aligned the raw sequences with Clustal W in BioEdit 7.0.9 by examining the chromatograms for polymorphic sites. MEGABLAST was used to check the identities of all the sequences against genomic references and nucleotide collections in the BOLD and GenBank databases, and conceptual amino acid translation was performed with the invertebrate mitochondrial criterion in MEGA 11 to detect possible *Numts* (nuclear copies of mtDNA fragments) [20]. A search for non-synonymous mutations, in-frame stop codons, and indels was also carried out to detect possible cryptic *Numts*. The Kimura two-parameter (K2P) distances between taxa were calculated in MEGA 11.

All the sequences were included in the phylogenetic reconstructions without pruning identical haplotypes to test the phylogenetic integrity of the species as identified using morphological characters. The phylogeny was reconstructed using the IQ-Tree method implemented in PhyloSuite 1.2.2 [21]. ModelFinder [22] was used to select the best fit model, with partition (edge-unlinked) implemented [23]. We then used the ultrafast bootstrap (1000 replications) function in the IQ-Tree to test the robustness of the tree.

### 2.4. Morphological Comparison

Specimens were spread for morphological comparison before molecular analysis based on habitus. The male and female forewing lengths were measured to 0.5 mm precision using a ruler. The whole abdomen was removed and placed into a 1.5 mL microcentrifuge tube and treated with 1 mL 10% sodium hydroxide (NaOH) solution for 1 h at 70 °C to digest the soft tissue. The treated abdomen was then neutralized with 2% acetic acid and dissected in a water-filled Petri dish under a stereomicroscope to remove residual tissues, scales, and hair. The genitalia were transferred to 80% glycerol for 12 h to render them transparent.

Habitus images were taken using a Canon 7D camera in conjunction with a Canon MP-E 65mm f/2.8 1–5X Macro Lens and a Canon MT-24EX Macro Twin Lite Flash as a light source. Images of the genitalia were taken using a Canon G9 camera mounted on an Olympus CX31 microscope under reflection or transmission lighting. Zerene Stacker (version 1.04) was used for image stacking. All images were further adjusted and annotated using Adobe Photoshop CS6. The dissected genital structures were stored in pure glycerol in a plastic centrifuge tube and labelled with detailed information of the specimens.

## 3. Results

### 3.1. Molecular Phylogenetic Analysis

**Figure 5:**
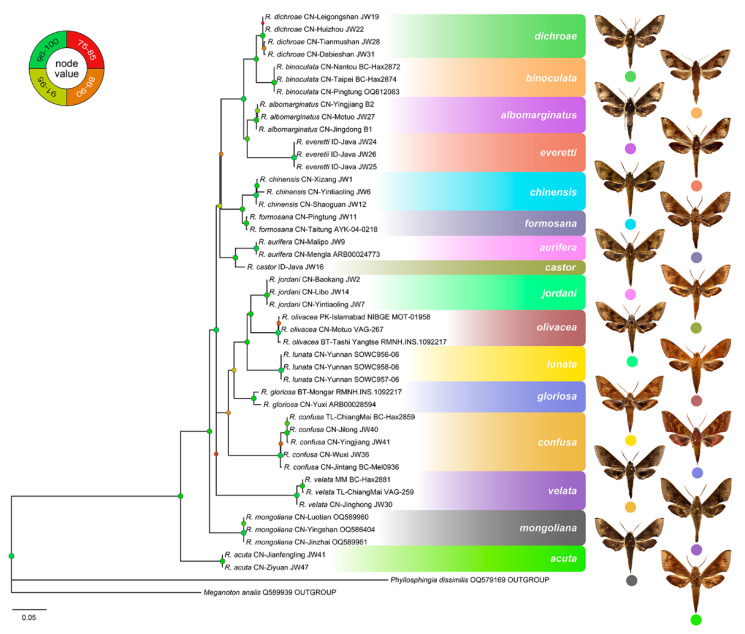
Maximum likelihood IQ-Tree phylogenetic tree of genus *Rhagastis* based on the DNA barcode sequences (*cox1*) and rooted with *Phyllosphingia dissimilis* and *Meganoton analis* as outgroups. Values at the nodes indicate bootstrap values.

**Figure 6:**
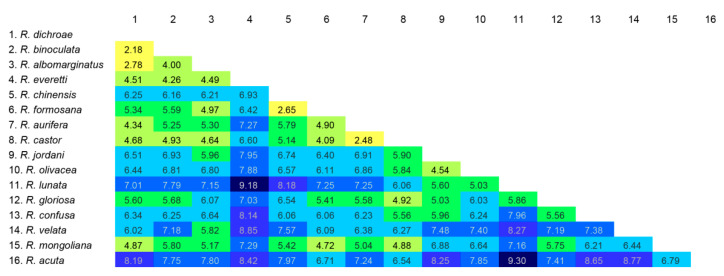
Kimura two-parameter (K2P) distances (in percentages) between all taxa of genus *Rhagastis* calculated from the DNA barcode sequences (*cox1*), with species identified as in the IQ-Tree tree in Figure 5.

ModelFinder selected the best fit model as TIM2+F+I+I+R2. The IQ-Tree reconstruction converged well, and the phylogenetic analysis recovered the 16 species of *Rhagastis* as monophyletic, with 10 species supported by maximal node values (Figure 5). The K2P genetic distances between taxa are shown in Figure 6.

The first clade consists of *R. dichroae* stat. nov. and *R. binoculata* as sister species, while the second clade consists of *R. everetii* stat. nov. and *R. albomarginatus*. The four above-mentioned species belong to the *albomarginatus* group. The third clade comprises *R. chinensi*s stat. nov. and *R. formosana* stat. nov. as sister species, while the fourth clade comprises *R. aurifera* stat. rev. and *R. castor*; these four species belong to the *castor* group. The fifth clade comprises *R. jordani* stat. rev., *R. olivacea*, and *R. lunata*, and is a sister species to *R. gloriosa* and *R. confusa*; these five species belong to the *olivacea* group. *R. velata* is closer to the *olivacea* group than to *R. mongoliana* and *R. acuta* (Figure 5). Given this molecular evidence, we propose the subspecies of *R. albomarginatus* and *R. castor* should be treated as good species.

### 3.2. Taxonomic Revision

#### 3.2.1. *Rhagastis albomarginatus* (Rothschild, 1894) [白肩天蛾]

*Metopsilus albomarginatus* Rothschild, 1894; Novit. Zool., 1: 78; **Type locality:** India, Assam, Khasia Hills.
  *Rhagastis albomarginatus nubilosa* Bryk, 1944; Arkiv för Zoologi, 8: 1–55 [24].

**Figure 7:**
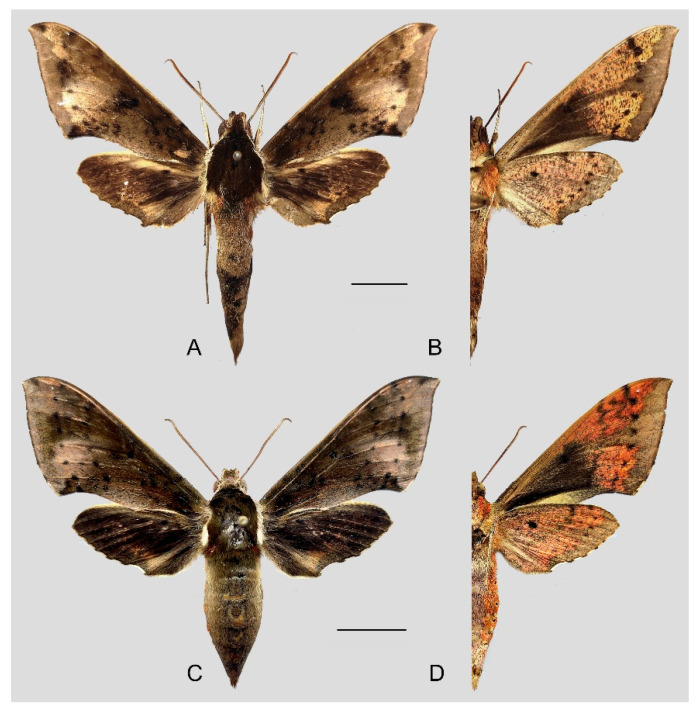
Photos of *Rhagastis albomarginatus*. (**A**,**B**) Male; Motuo, Xizang, China; (**C**,**D**) Female; Malipo, Yunnan, China. Scale bar = 10 mm.

**Diagnosis:** Male (Figure 7A,B): Head—grayish black; thorax—blackish gray with two creamy white stripes dorsally; abdomen—upper side black, lateral side with orange hair, underside grayish white. Forewingelongated, apex sharply pointed, outer margin protruding, distal portion of inner margin slightly concave; upper side—ground color dark gray–brown, middle area with black zigzag dotted lines and a black patch near the tornus, a yellow–white oval patch across the postmedian lines, a triangular deep gray patch near the apex, the submarginal area covered by a grayish patch, a discal spot near a large black patch; underside—yellow peppered with gray spots, the postmedian line a black dotted line, the large black patch at base connected to a grayish patch covered on the submarginal area with a gray thick line. Hindwing—dark brownish, tornus with a yellow-brown patch; underside—yellow peppered with gray spots, the postmedian line a black dotted line, the discal spot a black solid circle, the medial line a grayish zigzag line.

Female (Figure 7C,D): Similar to the male but with broader wings and a slightly paler and darker ground color and a more obvious grayish zigzag medial line on the underside of the forewing and hindwing.

**Figure 8:**
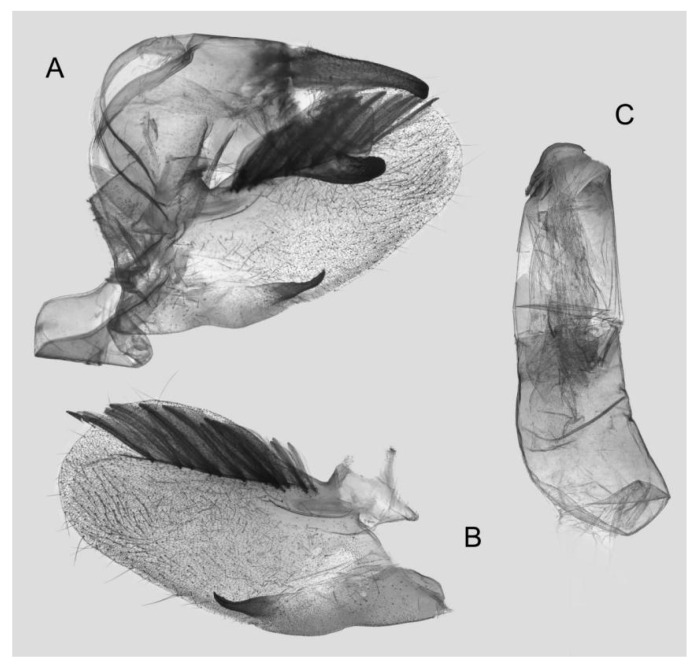
Male genitalia of *Rhagastis albomarginatus*, Jingdong, Yunnan, China. (**A**) Lateral view; (**B**) Left valve; (**C**) Phallus.

Male genitalia (Figure 8): The uncus and gnathos form a typical macroglossine “bird-beak” structure. Uncus straight, with a tiny apical hook. Gnathos slightly thicker than uncus; apex blunt. Valva rounded, with the basal part almost equaling the width of the terminal part; apex blunt. Sacculus slightly constricted and obviously curved upward apically into the harpe. Th phallus is short and straight, with the anterior lobe of the process ending in a slender transverse hook with apical spinules.

**Figure 9:**
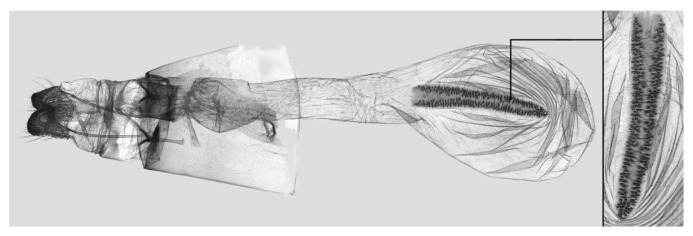
Female genitalia of *Rhagastis albomarginatus*, Malipo, Yunnan, China, with an enlarged signum on the right side.

Female genitalia (Figure 9): Anal papillae apophyses rounded. Lamella antevaginalis sclerotized and narrow; lamella postvaginalis blunt; ostial lobe short and wide. Ductus bursae tubular and membranous. Corpus bursae round; signum a long tongue-shaped ovoid sclerotized patch, with tiny spines on both sides.

**Distribution:** China (Yunnan, Guangxi, Xizang, Hainan), Nepal, Bhutan, India, Vietnam, Laos, Thailand.

#### 3.2.2. *Rhagastis dichroae* Mell, 1922 stat. nov. [姬白肩天蛾]

*Rhagastis albomarginatus dichroae* Mell, 1922; D. ent. Z., 1922: 120; **Type locality:** China, Kuangtung (Guangdong, China).
  *Rhagastis mongoliana centrosinaria* Chu & Wang, 1980; Acta Zootaxonomica Sinica, 5(4): 422 [25].

**Figure 10:**
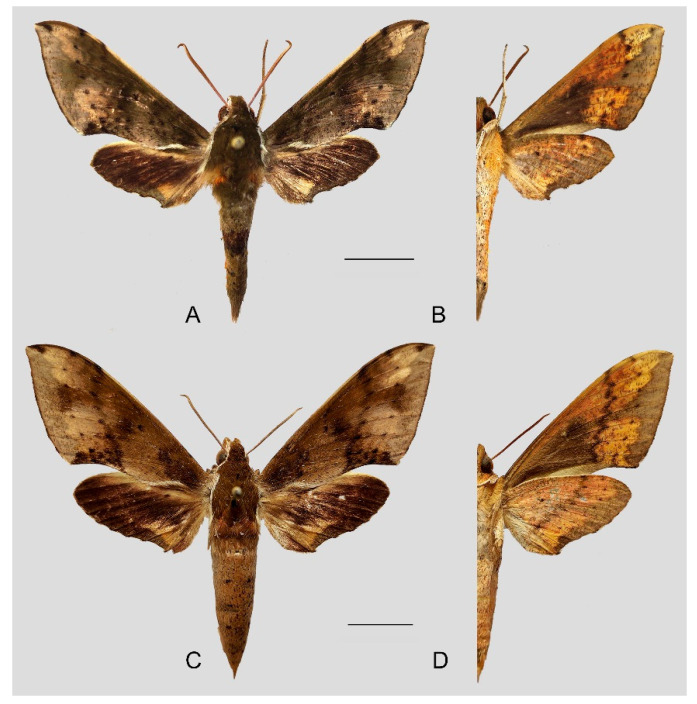
Photos of *Rhagastis dichroae* stat. nov. (**A**,**B**) Male; Shaoguan, Guangdong, China; (**C**,**D**) Female; Tianmushan, Zhejiang, China. Scale bar = 10 mm.

**Diagnosis:** Male (Figure 10A,B): Similar to *R. albomarginatus*, but the adult size is much smaller and the color tends to be dark green. Forewing narrower than that of *R. albomarginatus*; upper side oval patch across the postmedian lines smaller and faded; underside color tends to be orange–yellow; black solid circle on hindwing underside faded but still can be recognized.

Female (Figure 10C,D): Similar to the male but with broader wings and slightly paler ground color; grayish zigzag medial line on the underside of forewing and hindwing is more obvious.

**Figure 11:**
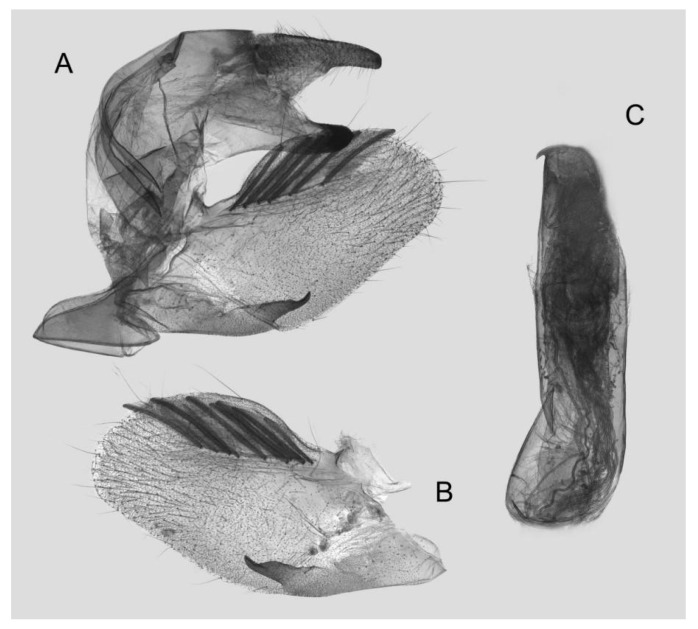
Male genitalia of *Rhagastis dichroae* stat. nov., Fanjingshan, Tongren, Guizhou, China. (**A**) Lateral view; (**B**) Left valve; (**C**) Phallus.

Male genitalia (Figure 11): Similar to those of *R. albomarginatus*, but the uncus and gnathos are shorter. Valva more rounded. Sacculus shorter and more curved than in *R. albomarginatus*. The phallus is straight, with the anterior lobe of the process ending in a slender transverse hook with fewer apical spinules.

**Figure 12:**
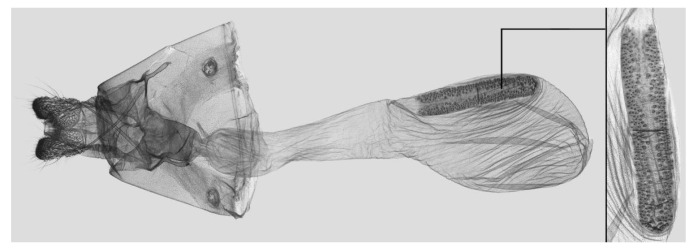
Female genitalia of *Rhagastis dichroae* stat. nov., Yingshan, Hubei, China, with an enlarged signum on the right side.

Female genitalia (Figure 12): Similar to those of *R. albomarginatus*, with a shorter and blunter signum and sparse tiny spines on both sides.

**Distribution:** Currently known to be found in China (Shaanxi, Chongqing, Hubei, Anhui, Zhejiang, Sichuan, Guizhou, Jiangxi, Hunan, Guangdong, and Guangxi).

#### 3.2.3. *Rhagastis everetti* Rothschild & Jordan, 1903 stat. nov. [长翅白肩天蛾]

*Rhagastis albomarginatus everetti* Rothschild & Jordan, 1903, Novit. Zool., 9: 799; **Type locality:** Malaysia, Sarawak, Kina Balu.
  *Rhagastis joiceyi* Clark, 1924; Proceedings of the New Zealand Zoological Club. 9:11–21 [26].

**Figure 13:**
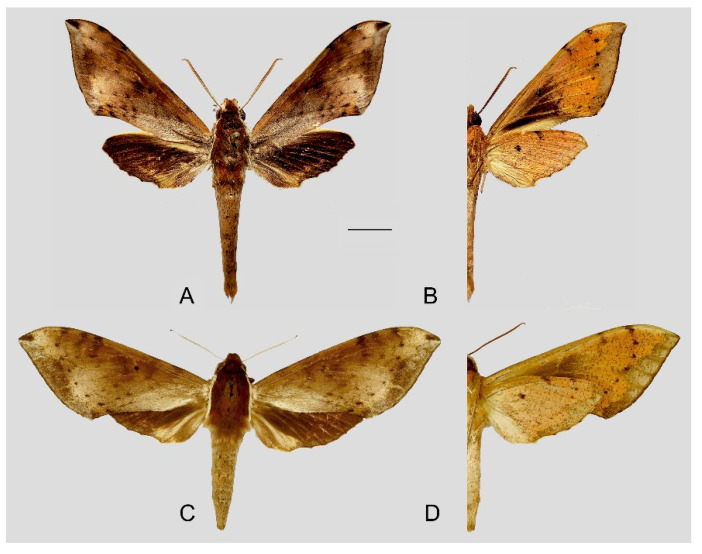
Photos of *Rhagastis everetti* stat. nov. (**A**,**B**) Male; Mt. Halimun, Java, Indonesia; (**C**,**D**) Female; Kina Balu, Malaysia. Scale bar = 10 mm. © The Trustees of the Natural History Museum, London, UK (downloaded from Kitching, I. Sphingidae Taxonomic Inventory. http://sphingidae.myspecies.info/. Available online: accessed on 31 January 2024).

**Diagnosis:** Male (Figure 13A,B): Similar to *R. albomarginatus*, but the adult size is larger and the color is paler. Forewing longer and narrower than that of *R. albomarginatus*; upper side color tends to be yellow-brown; faded black patch near the discal spot; underside color tends to be orange–yellow; black dotted postmedian lines faded but still can be recognized; the big black patch at base is disconnected from the grayish patch covering the submarginal area; grayish zigzag medial line on the underside of the forewing and hindwing almost faint, forming a short curved black stripe.

Female (Figure 13C,D): Similar to the male, but the wings are much broader and the ground color is paler.

**Figure 14:**
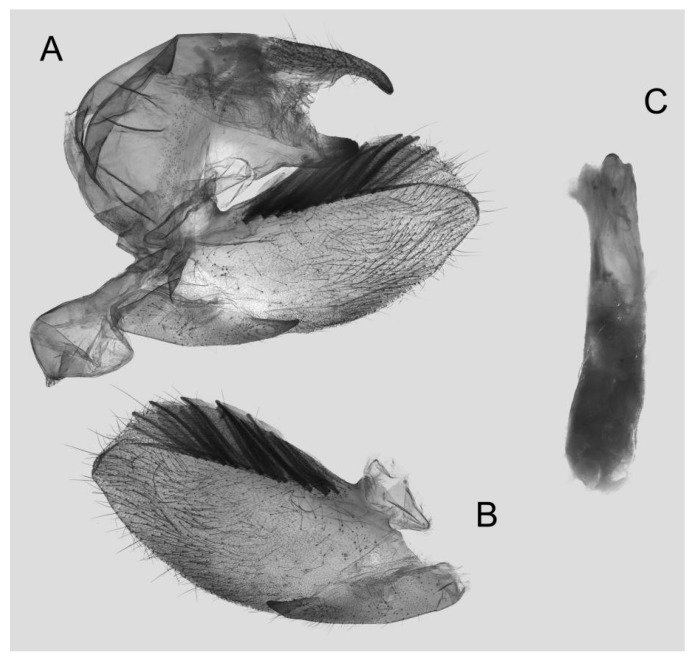
Male genitalia of *Rhagastis everetti* stat. nov., Mt. Halimun, Java, Indonesia. (**A**) Lateral view; (**B**) Left valve; (**C**) Phallus.

Male genitalia (Figure 14): Similar to those of *R. albomarginatus*, but the uncus is shorter and more curved. Valva slender, with the basal part wider than the terminal part. Sacculus much shorter and only slightly curved upward apically into the harpe. The phallus is slightly curved, with the anterior lobe of the process ending in a tiny sclerite without any apical spinules.

Female genitalia: Not examined due to the lack of material in this study.

**Distribution:** Malaysia (Peninsula), Borneo, Indonesia (Sumatra, Java).

#### 3.2.4. *Rhagastis binoculata* Matsumura, 1909 [双斑白肩天蛾]

*Rhagastis binoculata* Matsumura, 1909; Thous. Ins. Japan Suppl., 1: 39; **Type locality:** China, Taiwan, Puli-Wushe.
  *Rhagastis varia* Wileman, 1910; Entomologist, 43:288 [27].
  *Rhagastis elongata* Clark, 1937; Proceedings of the New England Zoological Club, 16: 27–39 [28].
  *Rhagastis albomarginatus sauteri* Mell, 1958; Deutsche Entomologische Zeitschrift, Berlin (N.F.), 5: 212 [29].

**Figure 15:**
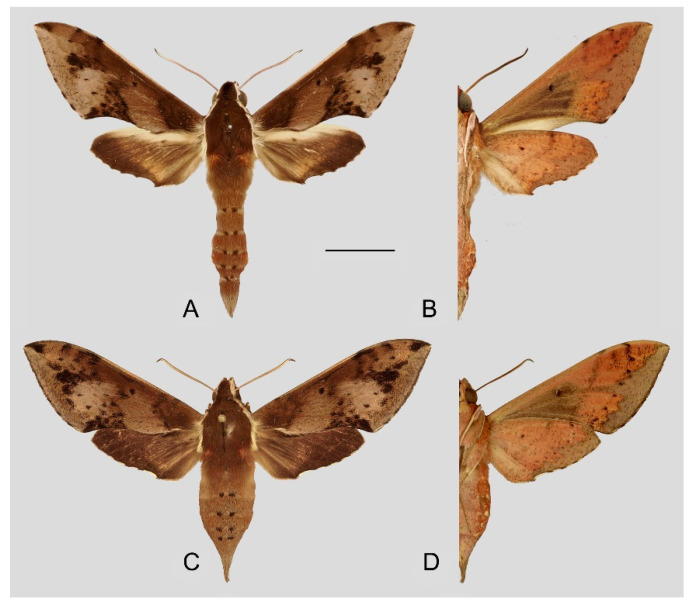
Photos of *Rhagastis binoculata*. (**A**,**B**) Male; Taipei, Taiwan, China; (**C**,**D**) Female; Taotuan, Taiwan, China. Scale bar = 10 mm. © The Trustees of the Natural History Museum, London, UK (downloaded from Kitching, I. Sphingidae Taxonomic Inventory. http://sphingidae.myspecies.info/. Available online: accessed on 31 January 2024).

**Diagnosis:** Male (Figure 15A,B): Very similar to *R. dichroae* stat. nov., mainly distinguishable by the pinkish-gray (yellowish-gray in *R. dichroae* stat. nov.) oval patch across the postmedian lines and deeper black spot on the forewing upper side. Color on the underside of forewing and hindwing tends to be orange-colored; patterns more blurred than in *R. dichroae* stat. nov. but still recognizable.

Female (Figure 15C,D): Similar to the male but with broader wings and slightly paler ground color; grayish zigzag medial line on the underside of the forewing and hindwing more obvious.

**Figure 16:**
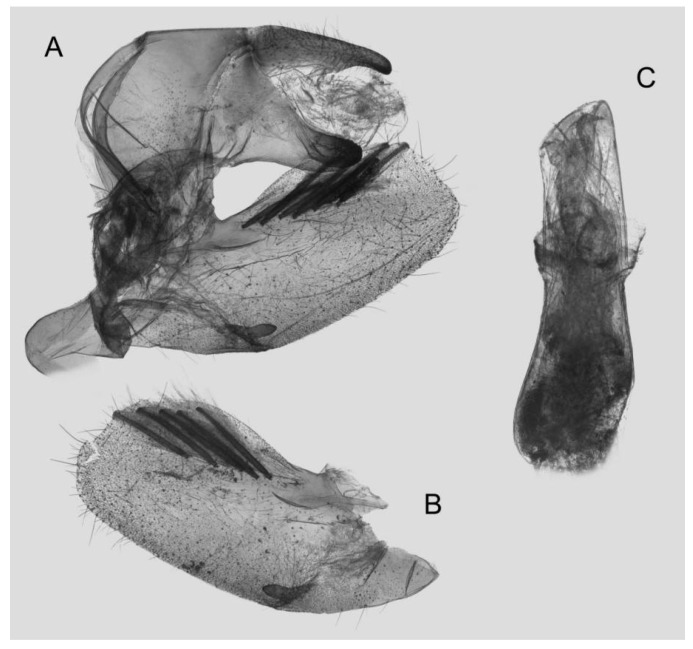
Male genitalia of *Rhagastis binoculata*, Donghe, Taitung, Taiwan, China. (**A**) Lateral view; (**B**) Left valve; (**C**) Phallus.

Male genitalia (Figure 16): Similar to that of *R. everetti* stat. nov., but the uncus and gnathos are shorter and thicker. Valva rounded, with the basal part narrower than the terminal part. Sacculus very short and little curved, and the apex is blunt.

Female genitalia: Not examined due to the lack of material in this study.

**Distribution:** Currently known to be from Taiwan Island of China.

#### 3.2.5. *Rhagastis castor* (Walker, 1856) [锯线白肩天蛾]

*Pergesa castor* Walker, 1856; List Spec. Lepid. Insects Colln Br. Mus., 8: 153; **Type locality:** Indonesia, Java.
  *Metopsilus aurantiacus* Rothschild, 1900; Novitates Zoologicae, 7: 274 [9].
  *Rhagastis aurifera sumatranus* Clark, 1924; Proceedings of the New Zealand Zoological Club. 9:11–21 [26].
  *Rhagastis javanica* Roepke, 1941; Verhandlingen der Koninklijke Nederlandsche Akademie van Wetenschappen, 1: 1–104 [30].

**Figure 17:**
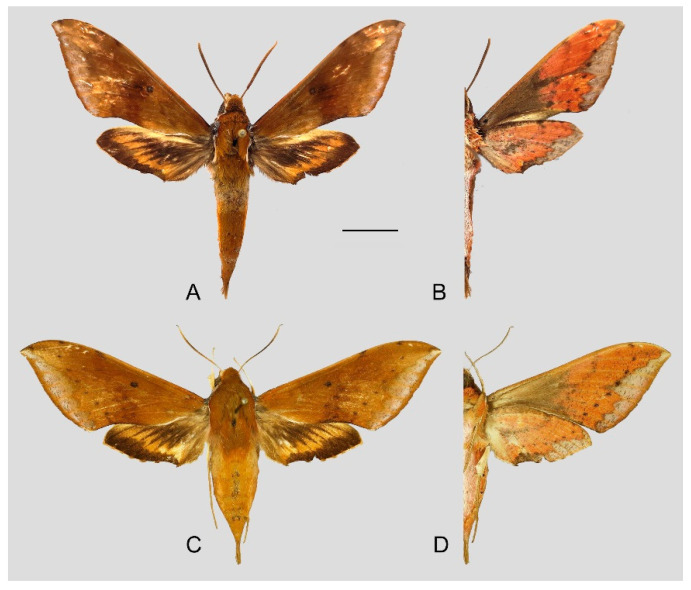
Photos of *Rhagastis castor*. (**A**,**B**) Male; Mt. Halimun, Java, Indonesia; (**C**,**D**) Female; Nongkodjad-jar, Java, Indonesia. Scale bar = 10 mm. © The Trustees of the Natural History Museum, London, UK (downloaded from Kitching, I. Sphingidae Taxonomic Inventory. http://sphingidae.myspecies.info/. Available online: accessed on 31 January 2024).

**Diagnosis:** Male (Figure 17A,B): Head—ochre-brownish with black hair; thorax—ochre-brownish with two creamy white stripes dorsally; abdomen—upper side brownish, each segment dotted with two black spots dorsally, lateral side with a long orange patch, underside grayish white. Forewing—elongated, apex sharply pointed, outer margin straight; upper side ground color ochre-brownish, middle area with black faded zigzag dotted lines, submarginal area covered with a silver–gray patch, outer margin yellow–brown, discal spot near a large deep brown patch; underside orange-reddish, postmedian line a black dotted line and medial line a grayish zigzag line, the deep gray at the base connected to a grayish patch covering the submarginal area with a gray slender line. Hindwing—black brownish, with a wide orange zigzag stripe across the middle area; underside orange-reddish, postmedian line a black dotted line, and medial line a grayish zigzag line, submarginal area covered by a gray patch.

Female (Figure 17C,D): Similar to the male but with broader wings and slightly paler and darker ground color; black dotted line and grayish zigzag medial line on the underside of forewing and hindwing more faded; grayish patch covering the submarginal area more obvious.

**Figure 18:**
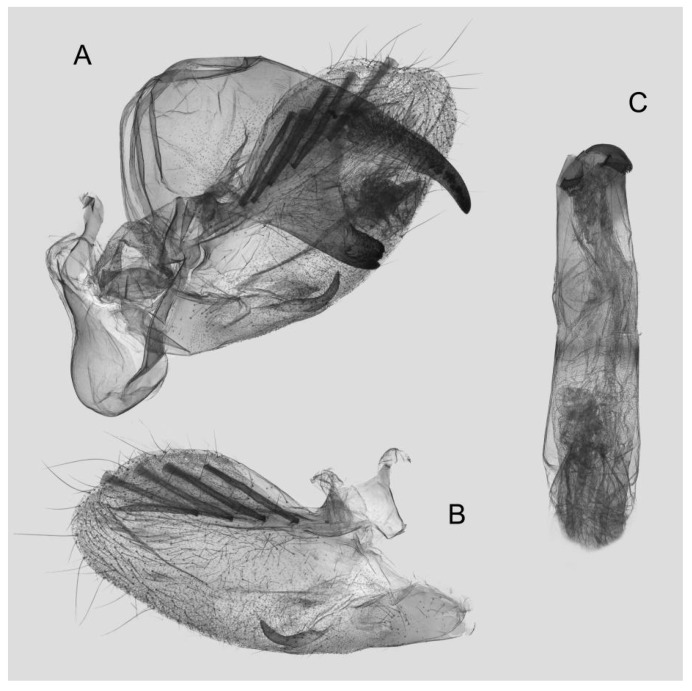
Male genitalia of *Rhagastis castor*, Mt. Halimun, Java, Indonesia. (**A**) Lateral view; (**B**) Left valve; (**C**) Phallus.

Male genitalia (Figure 18): The uncus and gnathos form a typical macroglossine “bird-beak” structure. Uncus straight and longer than the gnathos; curved with a tiny apical hook. Gnathos thicker than the uncus; apex blunt. Valva rounded, with the basal part almost narrower than the terminal part; apex blunt. Sacculus slightly constricted and obviously curved upward apically into the harpe. The phallus is long and straight, with the anterior lobe of the process ending in a thicker transverse hook with apical spinules on both sides.

**Figure 19:**
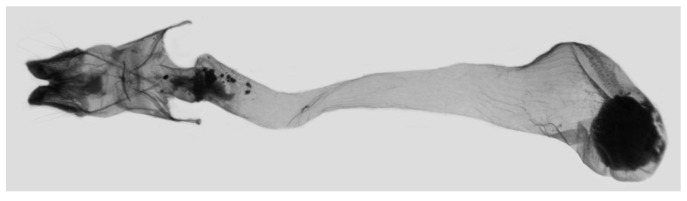
Female genitalia of *Rhagastis castor*, Java, Indonesia. © The Trustees of the Natural History Museum, London, UK (downloaded from Kitching, I. Sphingidae Taxonomic Inventory. http://sphingidae.myspecies.info/. Available online: accessed on 31 January 2024).

Female genitalia (Figure 19): Anal papillae apophyses sharp. Lamella antevaginalis sclerotized and narrow; lamella postvaginalis blunt; ostial lobe short and wide. Ductus bursae slender and long. Corpus bursae oblong; signum a long tongue-shaped ovoid sclerotized patch, with tiny spines on both sides.

**Distribution:** Malaysia (Borneo), Indonesia (Sumatra, Java).

#### 3.2.6. *Rhagastis aurifera* (Butler, 1875) stat. rev. [北印白肩天蛾]

*Pergesa aurifera* Butler, 1875; Proc. Zool. Soc. Lond., 1875: 7; **Type locality:** India, Sikkim.

**Figure 20:**
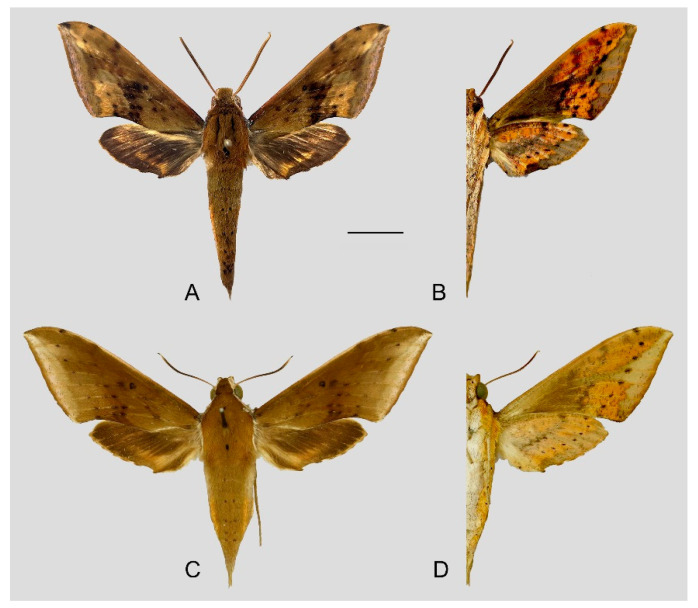
Photos of *Rhagastis aurifera* stat. rev. (**A**,**B**) Male; Malipo County, Yunnan, China; (**C**,**D**) Female; Shillong, Meghalaya, India. Scale bar = 10 mm. © The Trustees of the Natural History Museum, London, UK (downloaded from Kitching, I. Sphingidae Taxonomic Inventory. http://sphingidae.myspecies.info/. Available online: accessed on 31 January 2024).

**Diagnosis:** Male (Figure 20A,B): Similar to *R. castor*, but the adult size is smaller and the color tends to be greenish brown. Forewing—wider and straighter than that of *R. castor*; upper side ground color greenish brown, a yellowish oval patch across the postmedian lines, middle area with black zigzag dotted lines and a black patch near the discal spot, a triangular black patch near the apex, submarginal area covered by a pink-grayish patch; underside color and patterns deeper and more obvious than in *R. castor*. Hindwing—dark gray-black with a slender yellowish stripe across the middle area to the tornus; underside color and patterns deeper and more obvious than in *R. castor*, the grayish zigzag medial line dotted with some black spots.

Female (Figure 20C,D): Similar to the male but with broader wings and slightly paler ground color; patterns of the forewing and hindwing paler than in the male.

**Figure 21:**
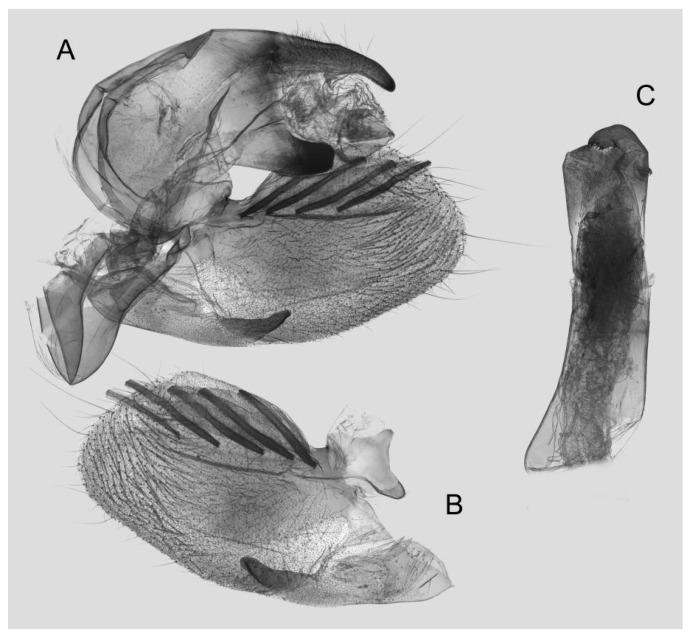
Male genitalia of *Rhagastis aurifera* stat. rev., Malipo, Yunnan, China. (**A**) Lateral view; (**B**) Left valve; (**C**) Phallus.

Male genitalia (Figure 21): Similar to that of *R. castor*, but the uncus is shorter. Valva more rounded. Sacculus thicker and apex obviously blunt. The phallus is shorter, with the anterior lobe of the process thicker than in *R. castor* and with gross apical serration.

Female genitalia: Not examined due to the lack of material in this study.

**Distribution:** China (Yunnan), Nepal, Bhutan, India, Thailand, Laos, Vietnam.

#### 3.2.7. *Rhagastis chinensis* Mell, 1922 stat. nov. [中华白肩天蛾]

*Rhagastis aurifera chinensis* Mell, 1922; D. ent. Zt. Berlin, 1922: 120; **Type locality:** SE. China.

**Figure 22:**
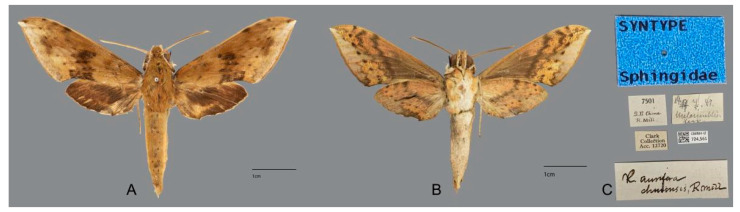
Syntype of *Rhagastis aurifera chinensis* Mell, 1922, SE. China, male. Photo by Vanessa Ver-decia, CMNH. (**A**) Upper side; (**B**) underside; (**C**) labels.

**Figure 23:**
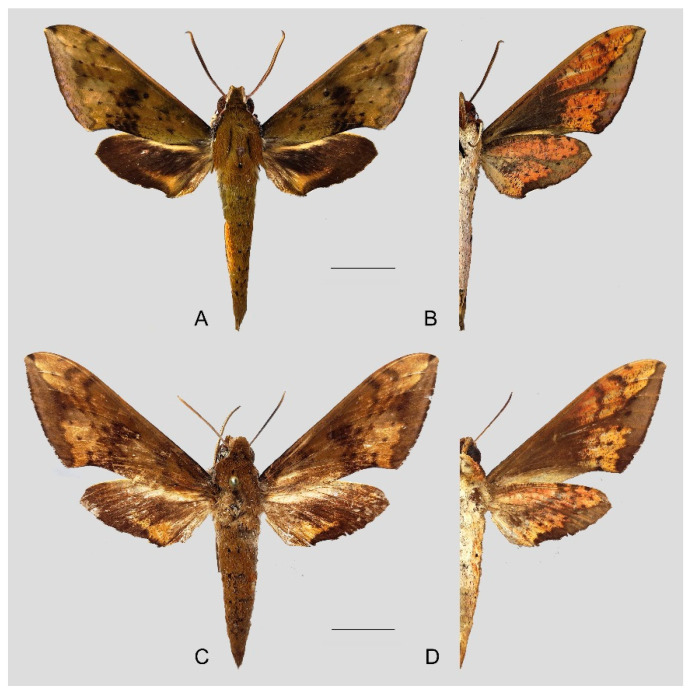
Photos of *Rhagastis chinensis* stat. nov. (**A**,**B**) Male; Yintiaoling, Wuxi, Chongqing, China; (**C**,**D**) Female; Lianzhou, Guangdong, China. Scale bar = 10 mm.

**Diagnosis:** Male (Figure 22 and Figure 23A,B): Very similar to *R. aurifera* stat. rev. but the forewing outer margin is more curved; oval patch across the postmedian lines on the forewing upper side less obvious than in *R. aurifera* stat. rev. The grayish zigzag medial line on hindwing underside is faded and without black spots.

Female (Figure 23C,D): Similar to the male but with broader wings and paler ground color; patterns of the forewing and hindwing more obvious than in the male.

**Figure 24:**
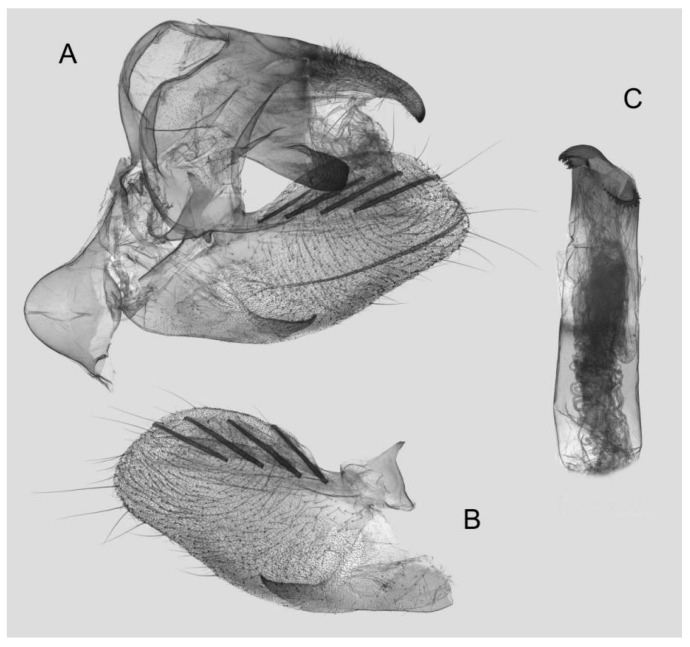
Male genitalia of *Rhagastis chinensis* stat. nov., Shaoguan, Guangdong, China. (**A**) Lateral view; (**B**) Left valve; (**C**) Phallus.

Male genitalia (Figure 24): Similar to that of *R. aurifera* stat. rev. Sacculus shorter and slightly curved upward apically into the harpe. The phallus is straighter than that of *R. aurifera* stat. rev., the anterior lobe of the process is longer, and one of the sides has gross and dense apical serration.

**Figure 25:**
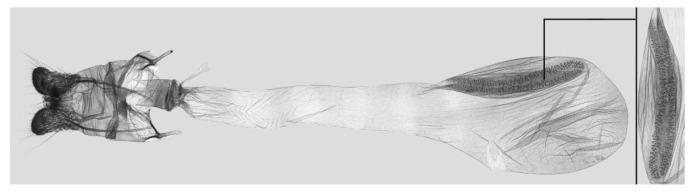
Female genitalia of *Rhagastis chinensis* stat. nov., Lianzhou, Guangdong, China, with an enlarged signum on the right side.

Female genitalia (Figure 25): Similar to that of *R. castor*. Anterior apophysis shorter and thicker; Ductus bursae shorter and wider. Corpus bursae more rounded; signum a long tongue-shaped ovoid sclerotized patch with tiny spines.

**Distribution:** China (Sichuan, Shaanxi, Chongqing, Zhejiang, Fujian, Jiangxi, Hunan, Guangdong, Guangxi, Yunnan, Xizang, Guizhou), Thailand, Laos, Vietnam.

#### 3.2.8. *Rhagastis formosana* Clark, 1925 stat. nov. [台湾白肩天蛾]

*Rhagastis aurifera formosana* Clark, 1925; Proc. New Engl. Zool. Club, 9: 37; **Type locality:** China, Taiwan, Nantou, Puli.

**Figure 26:**
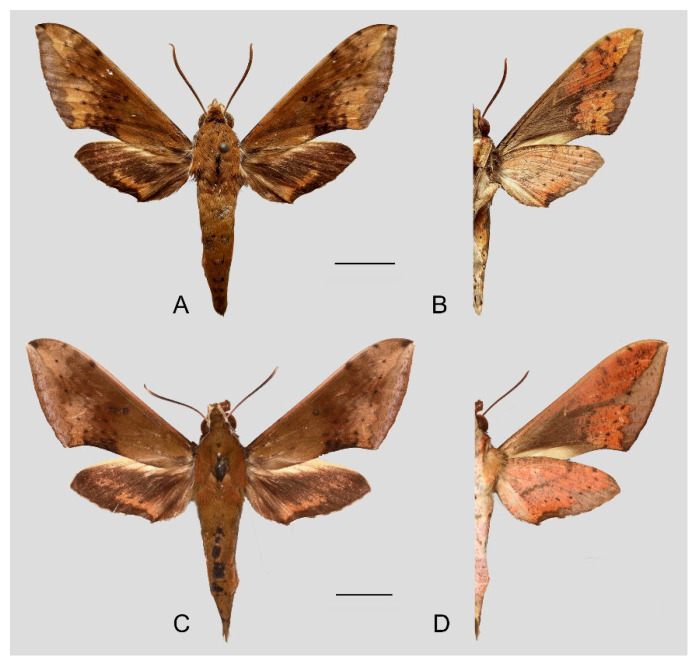
Photos of *Rhagastis formosana* stat. nov. (**A**,**B**) Male; Donghe, Taitung, Taiwan, China; (**C**,**D**) Female; Hualien, Taiwan, China. Scale bar = 10 mm.

**Diagnosis:** Male (Figure 26A,B): Very similar to *R. chinensis* stat. rev. but color and patterns paler.

Female (Figure 26C,D): Similar to the male but with broader wings and paler ground color; patterns of the forewing and hindwing more faded than in the male.

**Figure 27:**
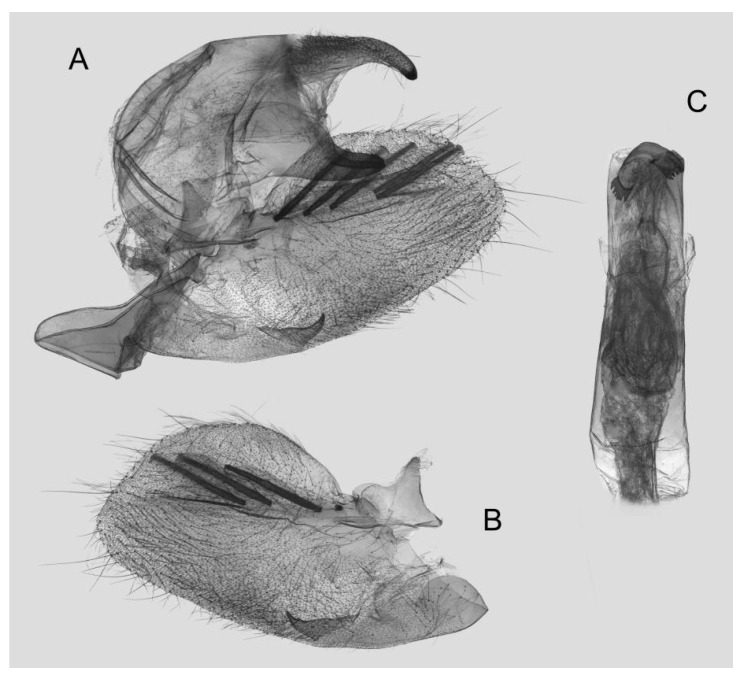
Male genitalia of *Rhagastis formosana* stat. nov., Donghe, Taitung, Taiwan, China. (**A**) Lateral view; (**B**) Left valve; (**C**) Phallus.

Male genitalia (Figure 27): Similar to that of *R. chinensis* stat. rev. Valve more rounded. Sacculus shorter and wider. The phallus is of medium length and straight, with the anterior lobe of the process strongly curved, and one of the sides with sparse apical serration.

**Figure 28:**
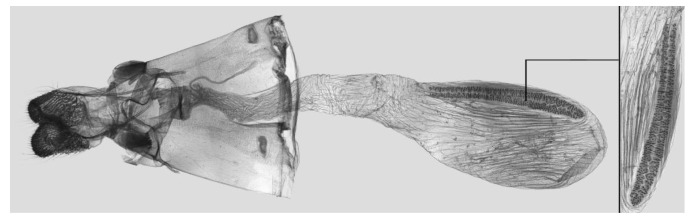
Female genitalia of *Rhagastis formosana* stat. nov., Hualien, Taiwan, China, with an enlarged signum on the right side.

Female genitalia (Figure 28): Similar to that of *R. chinensis* stat. rev. Anal papillae apophyses thicker; ostial lobe shorter; ductus bursae more curved and shorter. Corpus bursae elongated; signum shorter and wider than in *R. chinensis* stat. rev.

**Distribution:** Currently known to be from Taiwan Island of China.

#### 3.2.9. *Rhagastis jordani* Oberthür, 1904 stat. rev. [乔氏白肩天蛾]

*Rhagastis jordani* Oberthür, 1904; Bull. Soc. ent. Fr., 1904: 14; **Type locality:** China, Sichuan, Siao-lou.

**Figure 29:**
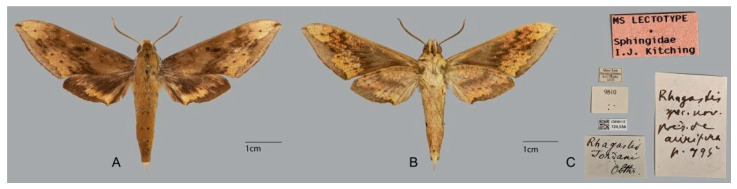
Lectotype of *Rhagastis jordani* Oberthür, 1904, Siao-Lou, Sichuan, China; male. Photo by Vanessa Verdecia, CMNH. (**A**) Upper side; (**B**) Underside; (**C**) Labels.

**Figure 30:**
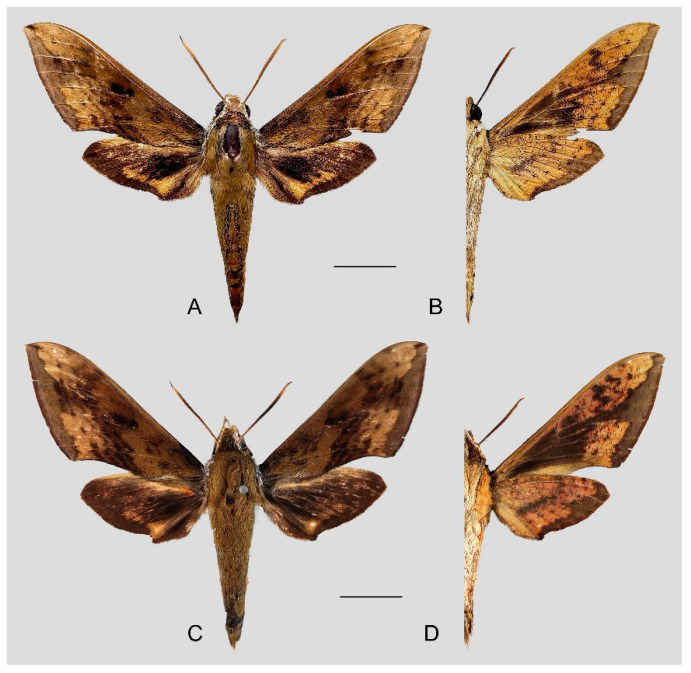
Photos of *Rhagastis jordani* stat. nov. (**A**,**B**) Male; Yintiaoling, Wuxi, Chongqing, China; (**C**,**D**) Male; Baokang, Xiangyang, Hubei, China. Scale bar = 10 mm.

**Diagnosis:** Male (Figure 29 and Figure 30A–D): Very similar to *R. chinensis* stat. rev. but the size is larger and the color tends to be more yellow. Forewing much wider and longer; outer margin more curved; oval patch across the postmedian lines and near the apex and black zigzag lines on the forewing upper side more obvious than in *R. chinensis* stat. rev.; the blackish zigzag medial line on forewing underside are much thicker.

Female: Similar to the male, but the wings are broader and the ground color is paler; patterns of the forewing and hindwing more faded than in the male.

**Figure 31:**
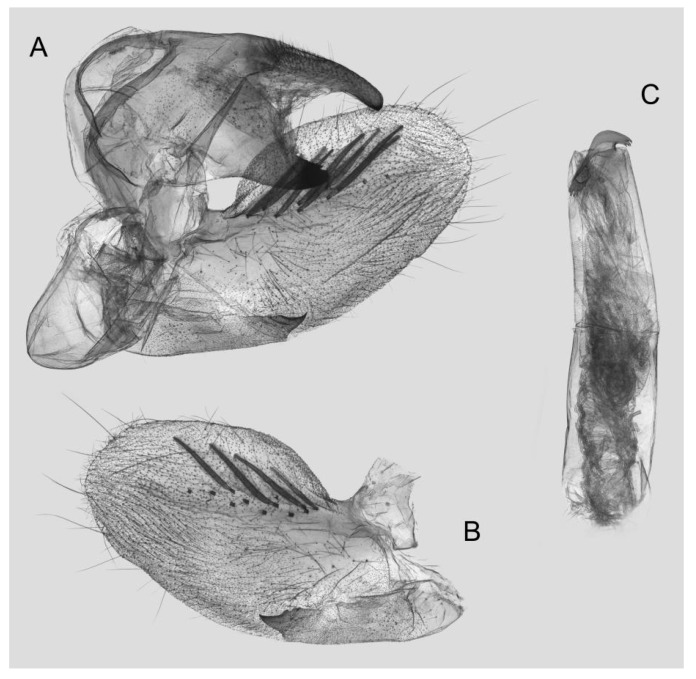
Male genitalia of *Rhagastis jordani* stat. rev., Yintiaoling, Wuxi, Chongqing, China. (**A**) Lateral view; (**B**) Left valve; (**C**) Phallus.

Male genitalia (Figure 31): Similar to that of *R. chinensis* stat. rev. Sacculus much shorter and apex bent upward apically like a spike. The phallus is longer and straighter, the anterior lobe of the process is slender, and one of the sides has sparse apical spines.

Female genitalia: Not examined due to the lack of material in this study.

**Distribution:** Currently known to be from China (Shaanxi, Chongqing, Hubei, Sichuan, Guizhou).

#### 3.2.10. *Rhagastis confusa* Rothschild & Jordan, 1903 [华西白肩天蛾]

*Rhagastis confusa* Rothschild & Jordan, 1903; Novit. Zool. 9 (suppl.): 793 (key), 795; **Type locality:** India, Meghalaya, Khasia Hills.
  *Rhagastis confusa chinensis* Clark, 1936; Proceedings of the New England Zoological Club, 15: 89 [31].
  *Rhagastis confusa peeti* Clark, 1936; Proceedings of the New England Zoological Club, 15: 90 [31].

**Figure 32:**
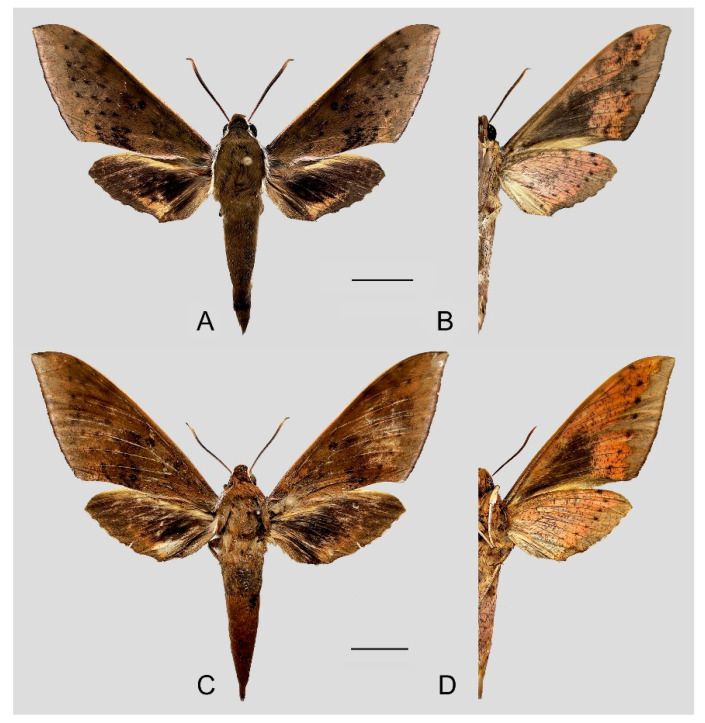
Photos of *Rhagastis confusa*. (**A**,**B**) Male; Yintiaoling Nature Reserve, Wuxi County, Chongqing, China; (**C**,**D**) Female; Xima Town, Yingjiang County, Yunnan, China. Scale bar = 10 mm.

**Diagnosis:** Male (Figure 32A,B): Similar to *R. chinensis* stat. rev. but of larger size and the color tends to be gray-brown. Forewing narrower and longer; outer margin straight; without oval patch across the postmedian lines; three black zigzag dotted lines on the middle of the forewing upper side. Ground color on the forewing and hindwing underside and the blackish zigzag medial line are more faded than in *R. chinensis* stat. rev.

Female (Figure 32C,D): Similar to the male but with broader wings; patterns of forewing and hindwing more faded than in the male.

**Figure 33:**
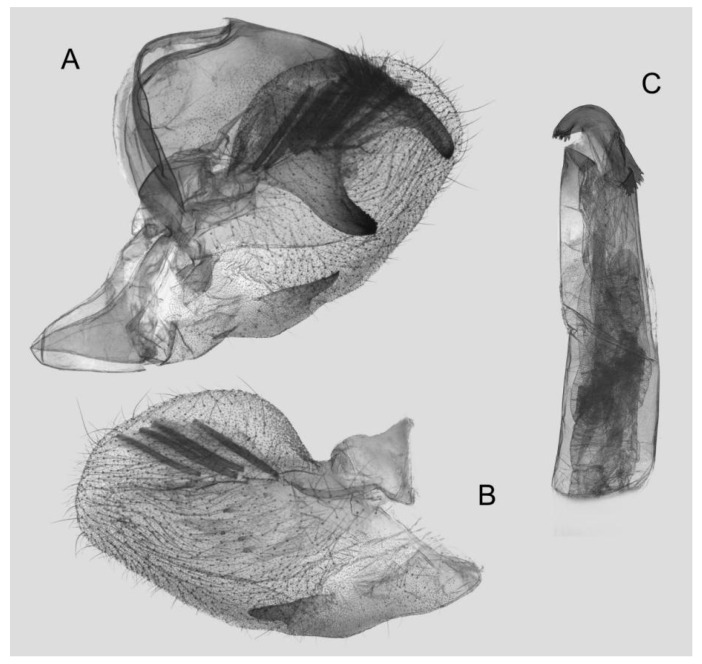
Male genitalia of *Rhagastis confusa*, Yintiaoling Nature Reserve, Wuxi County, Chongqing, China. (**A**) Lateral view; (**B**) Left valve; (**C**) Phallus.

Male genitalia (Figure 33): Similar to that of *R. chinensis* stat. rev. Gnathos curved and almost equal to the length of the uncus, with tiny tooth apically. Valva more rounded, with the basal part narrower than the terminal part; apex blunt. Sacculus straighter and apex bent a little upward apically. Phallus similar to that of *R. jordani* stat. rev.

**Figure 34:**
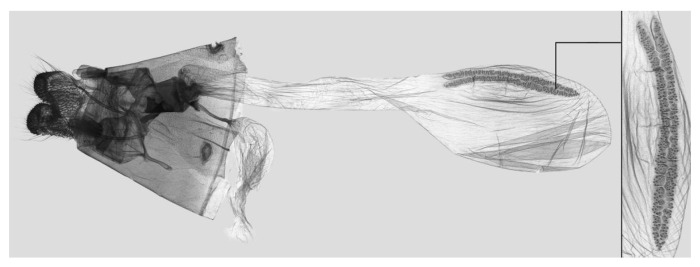
Female genitalia of *Rhagastis confusa*, Xima Town, Yingjiang County, Yunnan, China.

Female genitalia (Figure 34): Similar to *R. chinensis* stat. rev. Anal papillae wider. Ductus bursae shorter and slenderer. Corpus bursae oblong; signum slenderer than in *R. chinensis* stat. rev.

**Distribution:** China (Yunnan, Xizang, Guizhou, Sichuan, Chongqing, Shaanxi), Pakistan, Nepal, Bhutan, India, Myanmar, Vietnam.

#### 3.2.11. *Rhagastis velata* (Walker, 1866) [隐纹白肩天蛾]

*Pergesa velata* Walker, 1866; Insect Colln. Br. Mus., 35: 1853; **Type locality:** India, West Bengal, Darjeeling.
  *Theretra velata* Dudgeon, 1898, J. Bombay Nat. Hist. Soc., 11(2): 397 [31].

**Figure 35:**
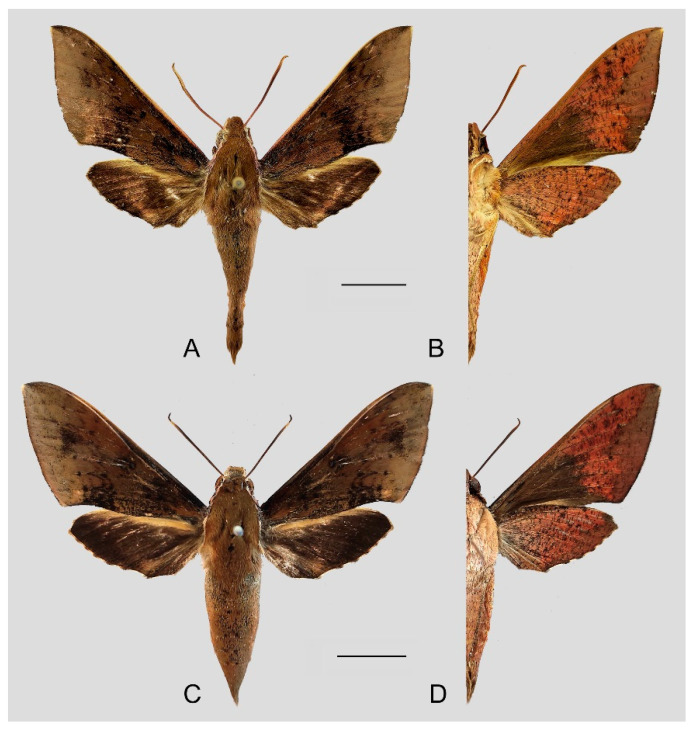
Photos of *Rhagastis velata*. (**A**,**B**) Male; Yanbian, Panzhihua, Sichuan, China. (**C**,**D**) Female; Xima, Yingjiang, Yunnan, China. Scale bar = 10 mm.

**Diagnosis:** Male (Figure 35A,B): Similar to *R. confusa* but much smaller in size; Forewing—apex protruding, with a black triangular patch; outer margin more curved; black zigzag lines on the forewing upper side expanding as a large black patch reaching the inner margin; underside color tends to be ochre, with many black fragmented spots dotted; the deep gray patch at the base is disconnected from the brown-grayish patch covering the submarginal area; patch color near hindwing tornus tends to be orange. The grayish zigzag medial line on the forewing and hindwing underside is much more faded than in *R. confusa*.

Female (Figure 35C,D): Similar to the male but with broader wings; patterns of the forewing and hindwing more faded than in the male.

**Figure 36:**
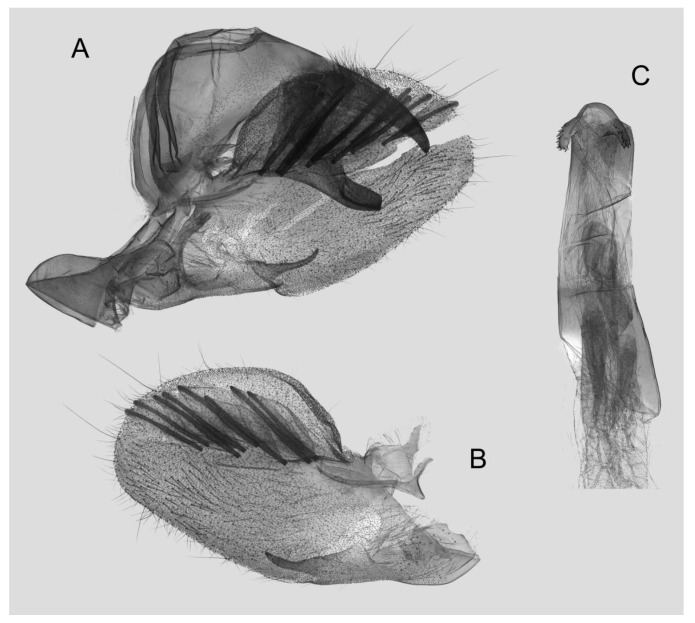
Male genitalia of *Rhagastis velata*, Tongren, Guizhou, China. (**A**) Lateral view; (**B**) Left valve; (**C**) Phallus.

Male genitalia (Figure 36): Similar to that of *R. confusa*. The uncus and gnathos are more curved and slenderer. Sacculus slender and curved upward apically like a spike. The phallus is longer and straighter, the anterior lobe of the process is slender, and the narrow side is sharper than in *R. confusa*.

**Figure 37:**
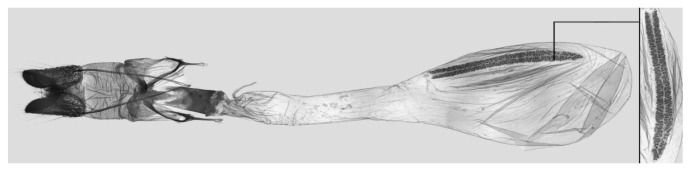
Female genitalia of *Rhagastis velata*, Xima, Yingjiang, Yunnan, China, with an enlarged signum on the right side.

Female genitalia (Figure 37): Similar to that of *R. confusa*. Anal papillae narrower. Posterior apophysis and anterior apophysis slender; ostial lobe longer. Corpus bursae oblong; signum slender and marked with denser tiny spines.

**Distribution:** China (Yunnan, Sichuan, Guizhou), Nepal, Bhutan, India, Thailand.

#### 3.2.12. *Rhagastis acuta* (Walker, 1856) [宽缘白肩天蛾]

*Zonilia acuta* Walker, 1856; Insect Colln. Br. Mus., 8: 195; **Type locality:** India.
  *Rhagastis hayesi* Diehl, 1982; Heterocera Sumatrana, 1: 71 [32].

**Figure 38:**
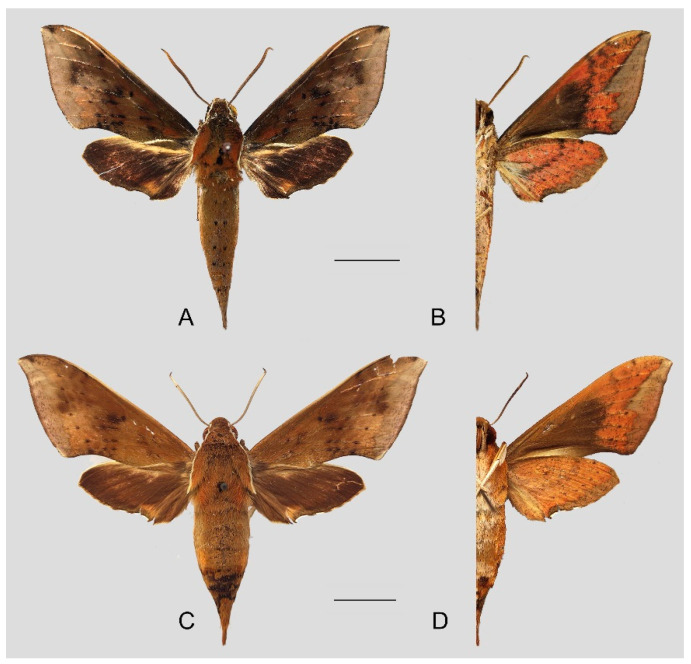
Photos of *Rhagastis acuta*. (**A**,**B**) Male; Ziyuan, Guangxi, China. (**C**,**D**) Female; Jianfengling, Ledong, Hainan, China. Scale bar = 10 mm.

**Diagnosis:** Male (Figure 38A,B): Similar to *R. velata* but ground color is with an ochreous hue; forewing wider and outer margin curved; tornus obviously concave near the inner margin; forewing base covered with ochre hair; the area between the outer margin to the postmedian lines yellowish-gray; a deep gray patch is marked on the submarginal line between M_1_ to M_3_. The black zigzag lines on the forewing upper side appear more obvious than in *R. velata*. The patch on the forewing underside submarginal tends to be gray and much wider than in *R. velata*; the blackish zigzag medial line on the forewing and hindwing underside is more obvious.

Female (Figure 38C,D): Similar to the male but with broader wings and paler ground color; patterns of the forewing and hindwing more faded than in the male.

**Figure 39:**
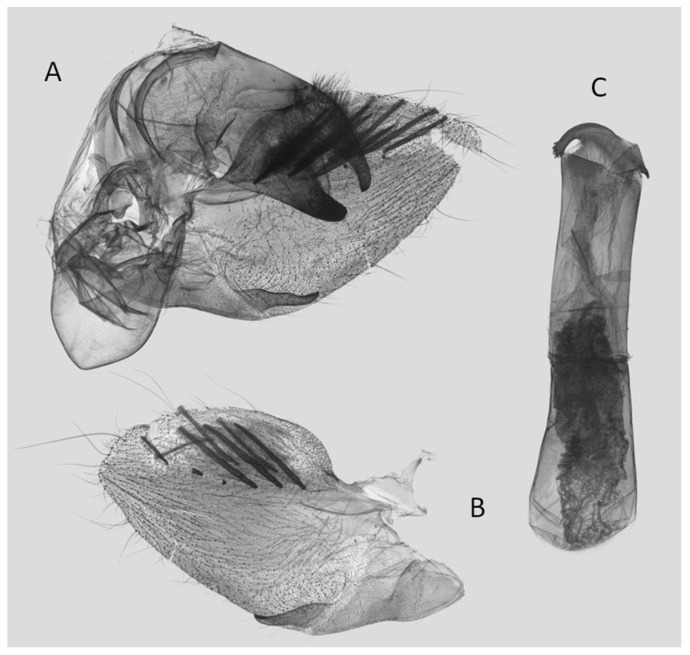
Male genitalia of *Rhagastis acuta*, Guangzhou, Guangdong, China. (**A**) Lateral view; (**B**) Left valve; (**C**) Phallus.

Male genitalia (Figure 39): Similar to that of *R. velata*. Uncus and gnathos shorter. Sacculus longer and more sinuous than in *R. velata*. The phallus is thicker, the anterior lobe of the process is slender, and one of the sides is contracted into a sharp tooth.

**Figure 40:**
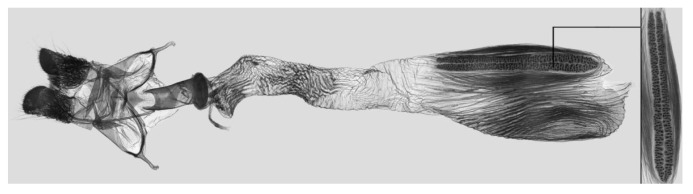
Female genitalia of *Rhagastis acuta*, Jianfengling, Ledong, Hainan, China, with the signum enlarged on the right side.

Female genitalia (Figure 40): Similar to that of *R. velata*. Anal papillae apophyses broader. Posterior apophysis and anterior apophysis shorter; anterior apophysis shorter with a small hook tip; ostial lobe more curved and shorter. Corpus bursae elongate; signum much wider than in *R. velata*.

**Distribution:** China (Guizhou, Yunnan, Guangdong, Guangxi, Hainan), India, Nepal, Bhutan, Bangladesh, Myanmar, Thailand, Cambodia, Vietnam, Philippines, Malaysia (Peninsular), Indonesia (Sumatra, Java).

#### 3.2.13. *Rhagastis mongoliana* (Butler, 1876) [蒙古白肩天蛾]

*Pergesa mongoliana* Butler, [1876]; Proc. Zool. Soc. Lond., 1875: 622; **Type locality:** Nankow Pass between China and Mongolia.
  *Rhagastis mongoliana pallicosta* Mell, 1922; Deutsche Entomologische Zeitschrift, Berlin, 1922(1): 120 [16].

**Figure 41:**
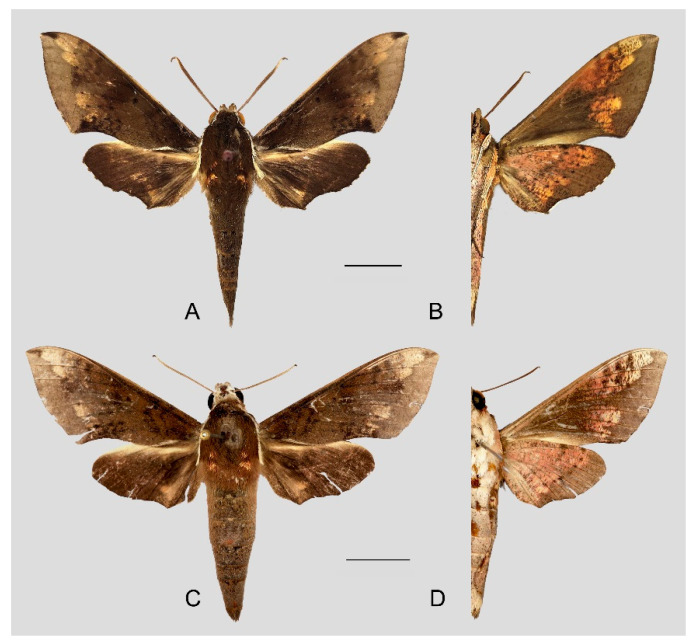
Photos of *Rhagastis mongoliana*. (**A**,**B**) Male; Benxi, Liaoning, China. (**C**,**D**) Female; Luo-tian, Hubei, China. Scale bar = 10 mm.

**Diagnosis:** Male (Figure 41A,B): Similar to *R. dichroae* stat. nov. but forewing broader; oval patch across the postmedian lines and near the apex on the forewing upper side and underside smaller and more faded than in *R. dichroae* stat. nov.; hindwing lacks the black discal spot.

Female (Figure 41C,D): Similar to the male but with broader wings and paler ground color; patterns of the forewing and hindwing more faded than in the male.

**Figure 42:**
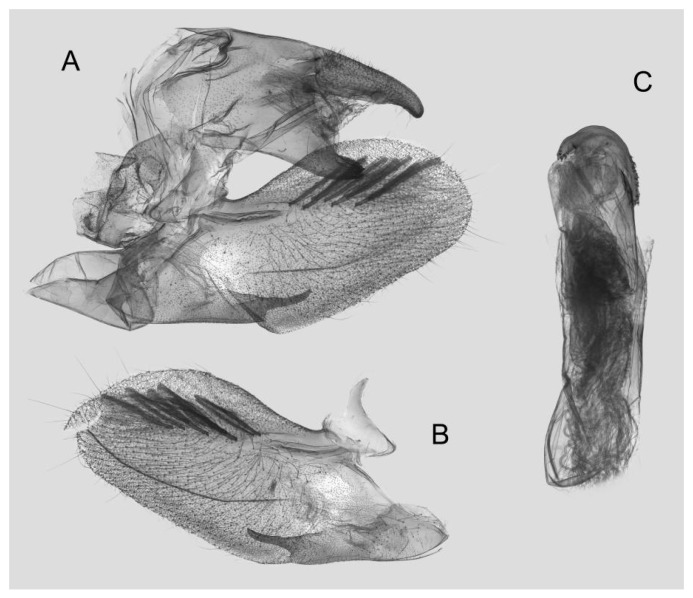
Male genitalia of *Rhagastis mongoliana*, Jinzhai, Anhui, China. (**A**) Lateral view; (**B**) Left valve; (**C**) Phallus.

Male genitalia (Figure 42): Similar to that of *R. acuta*. Sacculus slender and straighter. Phallus shorter and thicker; anterior lobe of the process is slender; and one of the sides extends longer than the other side and has intensive spinules around the edge.

**Figure 43:**
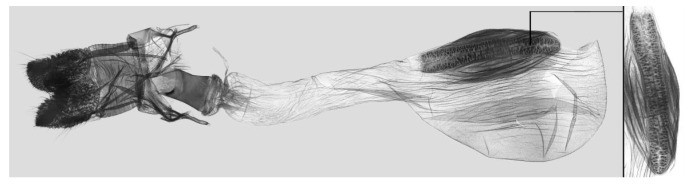
Female genitalia of *Rhagastis mongoliana*, Luotian County, Hubei, China, with the signum enlarged on the right side.

Female genitalia (Figure 43): Similar to that of *R. velata*. Anal papillae thicker. Ostium bursae shorter and wilder; ostial lobe straighter and broader. Corpus bursae more rounded; middle of the signum has sparser tiny spines than in *R. velata*; middle part narrower than both ends.

**Distribution:** China (Heilongjiang, Liaoning, Jilin, Beijing, Heibei, Inner Mongolia, Shanxi, Qinghai, Gansu, Anhui, Shaanxi, Shanghai, Zhejiang, Hubei, Sichuan, Chong-qing, Jiangxi, Hunan, Fujian, Guangdong, Guangxi, Taiwan, Hainan), Mongolia, North Korea, South Korea, Japan, Russia.

#### 3.2.14. *Rhagastis olivacea* (Moore, 1872) [青白肩天蛾]

*Pergesa olivacea* Moore, 1872; Proc. Zool. Soc. Lond., 1872: 567; **Type locality:** India, Himachal Pradesh, Simla.

**Figure 44:**
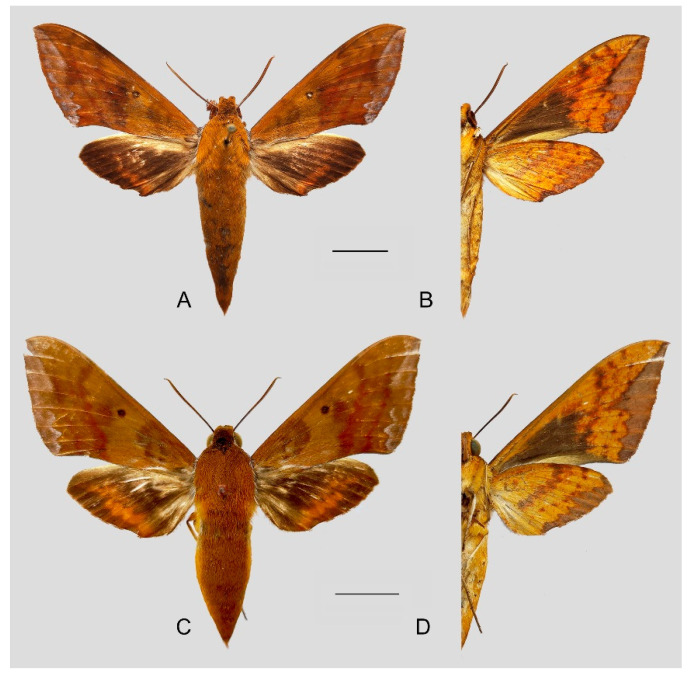
Photos of *Rhagastis olivacea*. (**A**,**B**) Male; Motuo, Xizang, China. (**C**,**D**) Female; Fuzhou, Jiangxi, China. Scale bar = 10 mm.

**Diagnosis:** Male (Figure 44A,B): Head—olive green; thorax—olive green with two creamy white stripes dorsally; abdomen—upper side olive green, lateral side with yellow hair, underside yellowish white. Forewing pattern similar to that in *R. acuta*; outer margin curved; upper side ground color olive green; a submarginal area covered by a silver–gray patch, with a waved edge; an ochre patch near the discal spot (a black hollow circle); sub-basal area marked with three brown curved lines and middle area with three ochre zigzag dotted lines; underside color orange; the patch covering the submarginal area tends to be brown-gray. Hindwing—black brownish, with a wide orange zigzag stripe across the middle area; underside orange-yellowish, with a black dotted postmedian line and a brown zigzag medial line.

Female (Figure 44C,D): Similar to the male but with broader wings; patterns of the forewing and hindwing wider and more obvious than in the male.

**Figure 45:**
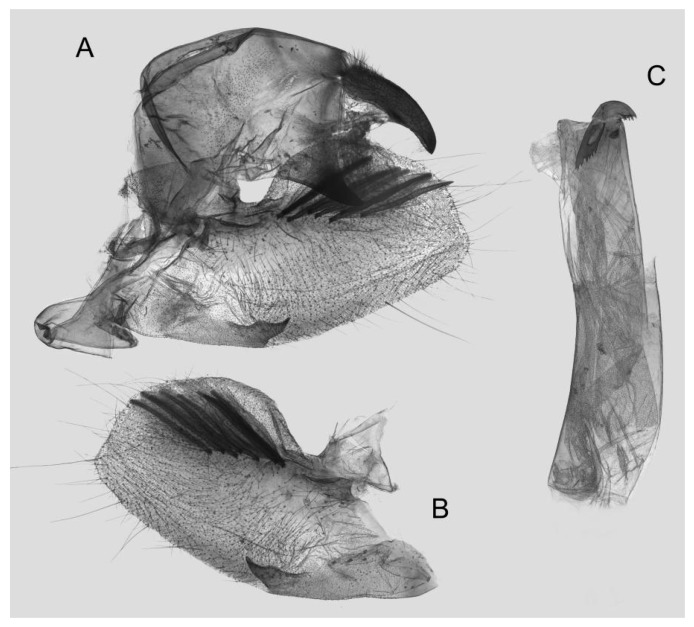
Male genitalia of *Rhagastis olivacea*, Shaoguan, Guangdong, China. (**A**) Lateral view; (**B**) Left valve; (**C**) Phallus.

Male genitalia (Figure 45): Similar to that of *R. jordani* stat. nov. Uncus and gnathos sharper. Sacculus slightly longer and tortuous, curving upward apically into the harpe. Phallus longer and straight, anterior lobe of the process ends in a thick transverse hook, and the narrow side is sharper than in *R. jordani* stat. nov.

**Figure 46:**
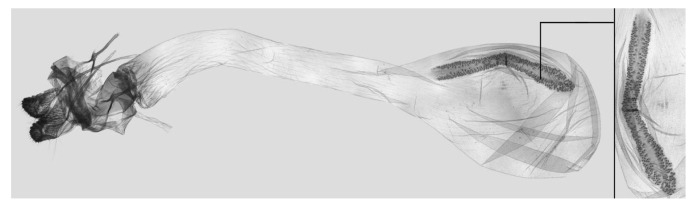
Female genitalia of *Rhagastis olivacea*, Motuo, Xizang, China, with the signum enlarged on the right side.

Female genitalia (Figure 46): Similar to that of *R. mongoliana*. Posterior apophysis and anterior apophysis much longer; sstium bursae broader and oval; ostial lobe much longer than in *R. mongoliana*. Corpus bursae round; signum much wider and buckling from the mid part.

**Distribution:** China (Zhejiang, Hubei, Sichuan, Yunnan, Xizang, Chongqing, Jiangxi, Hunan, Fujian, Guangdong, Guangxi, Hainan), Pakistan, India, Nepal, Bhutan, Myanmar, Thailand, Laos, Vietnam.

#### 3.2.15. *Rhagastis lunata* (Rothschild, 1900) [月纹白肩天蛾]

*Chaerocampa* [sic] *lunata* Rothschild, 1900; Novit. Zool., 7: 274; **Type locality:** India, Assam, Khasia Hills.
  *Rhagastis lunata sikhimensis* Rothschild & Jordan, 1903; Novit. Zool., 9 (Suppl.): 932 [1].
  *Rhagastis lunata gehleni* Bender, 1942; Mitteilungen der Münchener Entomologischen Gesellschaft, 32: 649 [33].
  *Rhagastis lunata yunnanaria* Chu & Wang, 1980; Acta Zootaxonomica Sinica, 5(4): 423 [25].
  *Rhagastis lunata yunnana* Chu & Wang, 1983; Iconographia Heterocerorum Sinicorum, 4: 406 [34].

**Figure 47:**
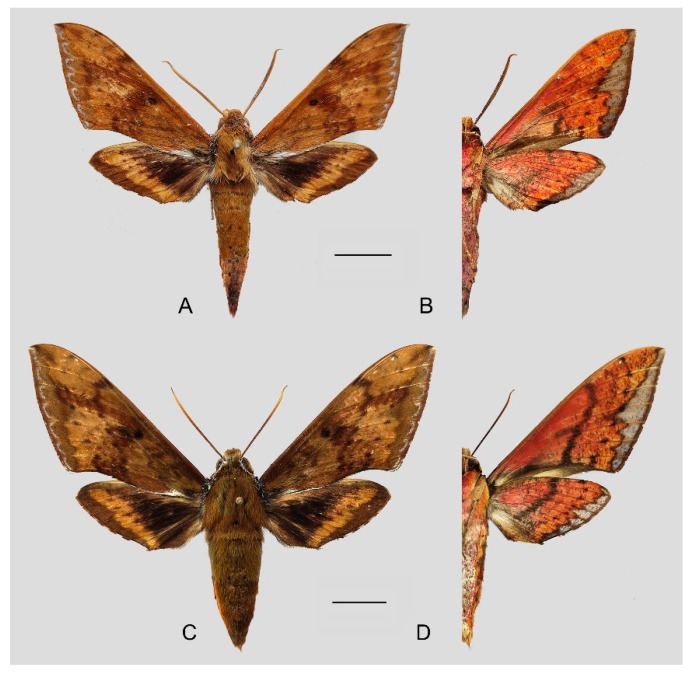
Photos of *Rhagastis lunata*. (**A**,**B**) Male; Baoshan, Yunnan, China. (**C**,**D**) Female; Zayu, Xizang, China. Scale bar = 10 mm.

**Diagnosis:** Male (Figure 47A,B): Similar to *R. olivacea* but ground color yellow-brown; abdomen with orange patch laterally; underside covered with rose and white hair. Forewing wider; apex protruding and with a brown patch; outer margin curved to tornus; forewing sub-basal curved lines and middle-area zigzag dotted lines deep brown; the area between outer margin to postmedian lines green-brown and connected to the discal spot with a brown stripe between M_1_ and M_2_; margin with a sliver gray wavy line across from the apex to tornus; underside covered in ochre. Hindwing black brownish, with a wide yellow zigzag stripe across the middle area. The patch covering the forewing and hindwing underside submarginal area tends to be silver–gray and narrower than in *R. olivacea*; the blackish zigzag medial line on underside appears more obvious.

Female (Figure 47C,D): Similar to the male but with broader wings and paler ground color; patterns of the forewing and hindwing more faded than in the male.

**Figure 48:**
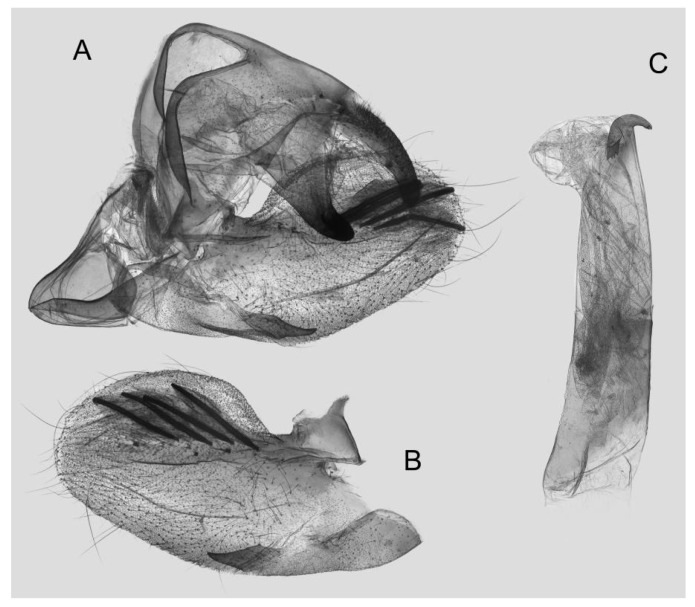
Male genitalia of *Rhagastis lunata*, Yingjiang, Yunnan, China. (**A**) Lateral view; (**B**) Left valve; (**C**) Phallus.

Male genitalia (Figure 48): Similar to that of *R. jordani* stat. nov. Uncus longer. Sacculus much longer and obviously sharper than in *R. jordani* stat. nov. Phallus thicker, anterior lobe of the process more curved and smoother.

**Figure 49:**
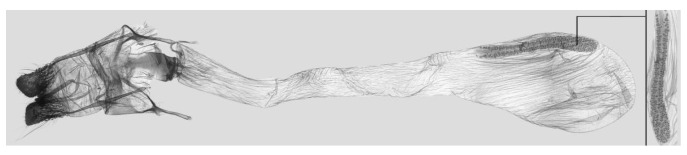
Female genitalia of *Rhagastis lunata*, Zayu, Xizang, China, with the signum enlarged on the right side.

Female genitalia (Figure 49): Similar to that of *R. olivacea*. Ductus bursae slender. Corpus bursae oblong; signum long tongue-shaped ovoid and not bucked as in *R. olivacea*.

**Distribution:** China (Yunnan, Xizang), India, Nepal, Bhutan, Myanmar, Thailand, Laos, Vietnam.

#### 3.2.16. *Rhagastis gloriosa* (Butler, 1875) [玫红白肩天蛾]

Pergesa gloriosa Butler, 1875; Proc. Zool. Soc. Lond., 1875: 246; **Type locality:** India, West Bengal, Darjiling.
  *Rhagastis gloriosa orientalis* Bryk, 1944; Arkiv för Zoologi, 8: 1–55 [24].
  *Rhagastis yunnanaria* Chu & Wang, 1980; Acta Zootaxonomica Sinica, 5(4): 422 [25].

**Figure 50:**
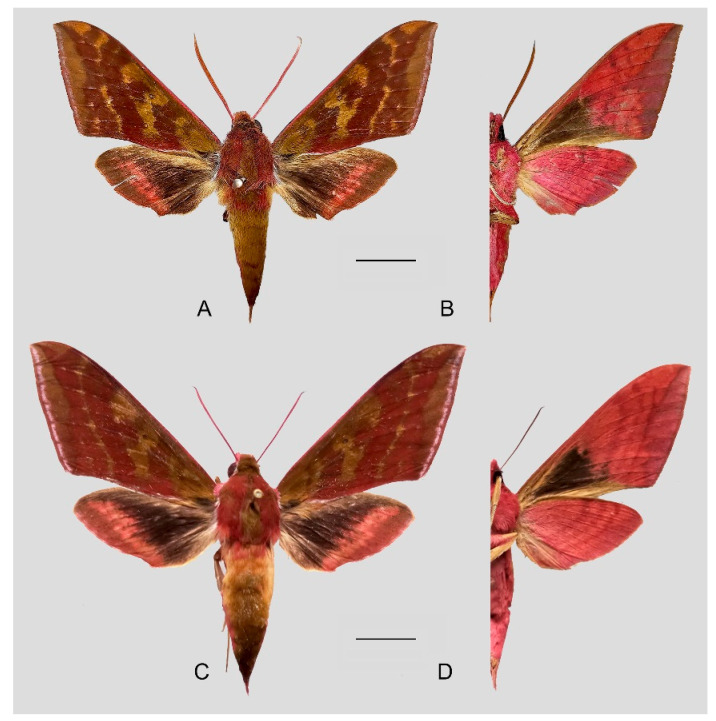
Photos of *Rhagastis gloriosa*. (**A**,**B**) Male; Pu’er, Yunnan, China. (**C**,**D**) Female; Motuo, Xizang, China. Scale bar = 10 mm.

**Diagnosis:** Male (Figure 50A,B): Similar to *R. lunata* but ground color red–ochre; thorax—red–ochre with two pink stripes and an olive green patch dorsally; abdomen–upper side olive green, lateral and underside rose. Forewing—wider; ground color olive green with three wide red–ochre stripes; a large red–ochre patch near the discal spot; margin with a silver–rose wavy line across from the apex to the tornus; forewing upper side rose, with three red-ochre zigzag lines; the patch covering the submarginal tends to be rose-brown. Hindwing—black brownish, with a wide rose zigzag stripe across the middle area, submarginal color olive green and rose; underside rose and with three faded rose–brown curved stripes.

Female (Figure 50C,D): Similar to the male but with broader wings and darker ground color; patterns of the forewing and hindwing broader than in the male.

**Figure 51:**
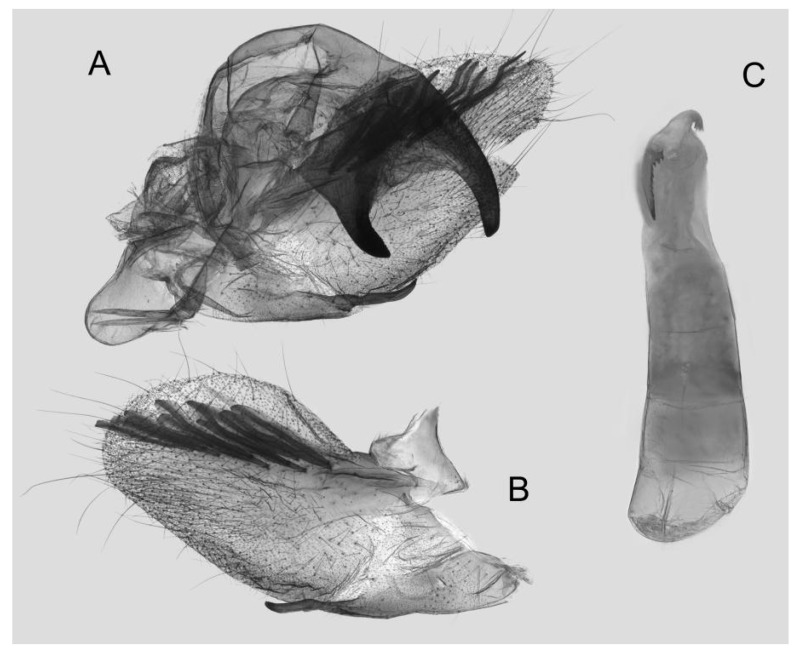
Male genitalia of *Rhagastis gloriosa*, Weixi, Yunnan, China. (**A**) Lateral view; (**B**) Left valve; (**C**) Phallus.

Male genitalia (Figure 51): Similar to that of *R. lunata*. Uncus longer and gnathos sharper. Sacculus much longer and wavy, curving upward apically like a spike. Phallus shorter, anterior lobe of the, process slender and the narrower side extends longer than the other side, appearing as a sharp harpe hook with few serrations.

**Figure 52:**
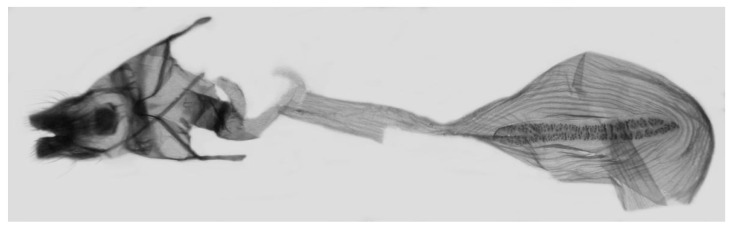
Female genitalia of *Rhagastis gloriosa*, Darjiling, India. © The Trustees of the Natural History Museum, London, UK (downloaded from Kitching, I. Sphingidae Taxonomic Inventory. http://sphingidae.myspecies.info/. Available online: accessed on 31 January 2024).

Female genitalia (Figure 52): Similar to that of *R. olivacea*. Anal papillae apophyses thinner; ostial lobe short and cylindrical. Ductus bursae shorter and more curved than in *R. olivacea*. Corpus bursae oval; signum long and tongue-shaped.

**Distribution:** China (Yunnan, Xizang), India, Nepal, Bhutan, Myanmar, Thailand, Vietnam.

### 3.3. Morphological Differences

**Figure 53:**
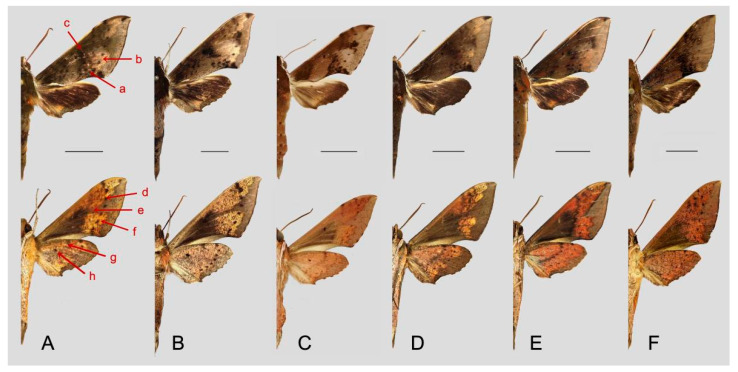
Morphological comparison of some similar species in genus *Rhagastis* of China (**A**). *R. dichroae* stat. nov., Shaoguan, Guangdong, China; male; (**B**). *R. albomarginatus*, Yingjiang, Yunnan, China; male; (**C**). *R. binoculata*, Taipei, Taiwan, China; male; (**D**). *R. mongoliana*, Benxi, Liaoning, China; male; (**E**). *R. acuta*, Ziyuan, Guangxi, China; male; (**F**). *R. velata*, Panzhihua, Sichuan, China; male. Upper side on the first row; underside on the second row; scale bar = 10 mm.

**Figure 54:**
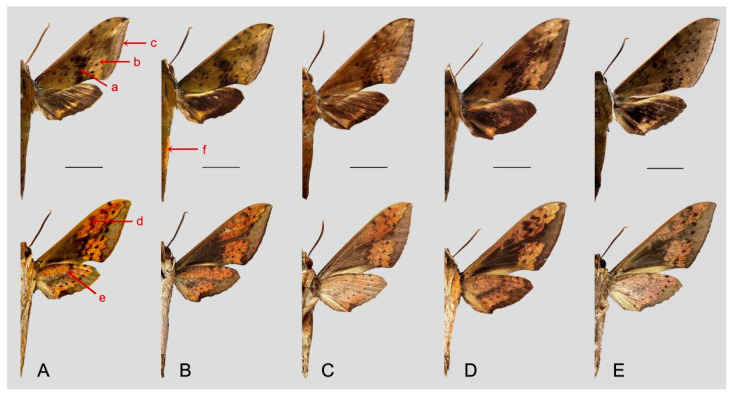
Morphological comparison of other similar species of genus *Rhagastis* of China (**A**). *R. aurifera* stat. rev., Malipo, Yunnan, China; male; (**B**). *R. chinensis* stat. nov., Wuxi, Chongaqing, China; male; (**C**). *R. formosana* stat. nov., Taitung, Taiwan, China; male; (**D**). *R. jordani* stat. rev., Baokang, Hubei, China; male; (**E**). *R. confusa*, Wuxi, Chongqing, China; male. Upper side on the first row; under-side on the second row; scale bar = 10 mm.

Comparison of the wing morphology of some of the similar species in genus *Rhagastis* of China showed constant differences (Figure 53 and Figure 54). In Figure 53, the following characters are important in separating these similar species: (1) the middle area of the forewing with black zigzag dotted lines and a black patch near the tornus in all species; only *R. velata* has a heavy black pattern reaching the inner margin (a); (2) the oval patch across postmedian lines on the forewing upper side in *R. dichroae* stat. nov. is smaller than that in *R. albomarginatus* the oval patch in *R. binoculata* is pinkish-gray rather than yellowish-white in *R. dichroae* stat. nov. and *R. albomarginatus*, and the oval patches in *R. mongoliana*, *R. acuta*, and *R. velata* are much smaller or absent (b); (3) the discal spots of *R. dichroae* stat. nov. and *R. albomarginatus* have a large black patch but are smaller or faded in other species (c); (4) on the forewing and hindwing undersides, the black dotted line on the postmedian lines is obvious in *R. dichroae* stat. nov. and *R. albomarginatus* but is faded in other species (d); (5) the yellow oval patch across the postmedian lines on the forewing underside is clear in *R. dichroae* stat. nov. and *R. albomarginatus* but is a smaller orange-yellowish patch in *R. mongoliana*; the forewing and hindwing are orange-reddish in *R. binoculata*, *R. acuta*, and *R. velata*, while there are many black fragmented spots in *R. velata* (f); (6) the large black patch on the base of the forewing connected to the grayish patch covering the submarginal area (this gray patch is much broader in *R. acuta*) is a thick grayish line in *R. dichroae* stat. nov., *R. albomarginatus*, and *R. mongoliana*, but this is faded in *R. binoculata* and disappears in *R. acuta* and *R. velata* (e); (7) the discal black spot on the hindwing in *R. dichroae* stat. nov., *R. albomarginatus*, and *R. binoculata* is absent in other species (h); (8) the gray median line on the hindwing zigzags in *R. acuta* and *R. mongoliana* but is faded in other species, even disappearing in some individuals of *R. velata* (g).

The following characteristics are important in separating the five remaining similar species (Figure 54): (1) both sides of the abdomen with an orange-yellowish patch in the *castor* group species (*R. aurifera* stat. rev., *R. chinensis* stat. nov., and *R. formosana* stat. nov.); the other two species (*R. jordani* stat. rev. and *R. confusa*) lack this abdominal characteristic (f); (2) the middle area of the forewing with heavy black zigzag dotted lines and a patch in all species except for *R. confusa*, which only has three clear and straight black dotted lines (a); (3) the oval yellowish patch across the postmedian lines on the forewing upper side appears in all species except in *R. confusa*, which is uniformly covered by brown-grayish scales (b); (4) the forewing of *R. aurifera* stat. rev. is narrower and longer than in *R. chinensis* stat. nov. and *R. formosana* stat. nov., the forewing of *R. jordani* stat. rev. is much broader than in other species, and the forewing of *R. confusa* is much longer than in other species (c); (5) the blackish zigzag median line on the forewing is thicker and more obvious in *R. aurifera* stat. rev. and *R. jordani* stat. rev. than in other species (d); (6) the grayish zigzag median line on the hindwing has more obvious black spots in *R. aurifera* stat. rev. than in other species, especially in *R. chinensis* stat. nov. (e).

### 3.4. Biological Notes and Ecological Record

**Figure 55:**
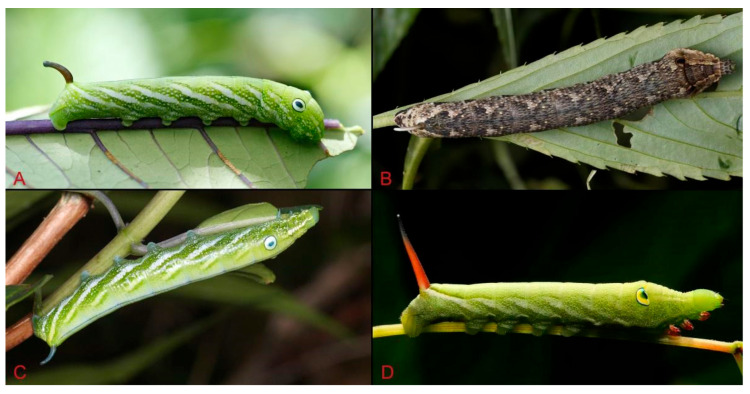
Larvae of genus *Rhagasti*s from China. (**A**). *R. dichroae* stat. nov., Shenzhen, Guangdong, China; (**B**). *R. mongoliana*, Chizhou, Anhui, China; (**C**). *R. binoculata*, Taipei, Taiwan, China; (**D**). *R. olivacea*, Nanling, Guangdong, China.

Larvae of *Rhagastis* species (Figure 55) have been recorded feeding on various plants. *R. albomarginatus* is recorded as feeding on species of *Dichroa*, *Hydrangea*, and *Vitis*; *R. dichroae* stat. nov. feeds on *Vernicia montana* and *Dichroa febrifuga*; *R. binoculata* feed on *Hydrangea chinensis*; *R. aurifera* stat. rev. is recorded as feeding on *Amorphophallus* and *Vitis*; *R. formosana* stat. nov. feeds on *Saurauia tristyla*, *H. chinensis*, and *Tetrastigma formosanum*; *R. confusa* is recorded as feeding on *Vitis*; *R. velata* is recorded as feeding on *Arisaema* and *Amorphophallus*; R. olivacea is recorded as feeding on *Impatiens*; *R. mongoliana* is recorded as feeding on various plants, such as *Impatiens walleriana*, *I. balsamina*, *Arisaema ringens*, *Psychotria serpens*, *V. amurensis*, *Cayratia japonica*, *Zantedeschia aethiopica*, and *Parthenocissus tricuspidata* [35,36,37,38,39].

**Figure 56:**
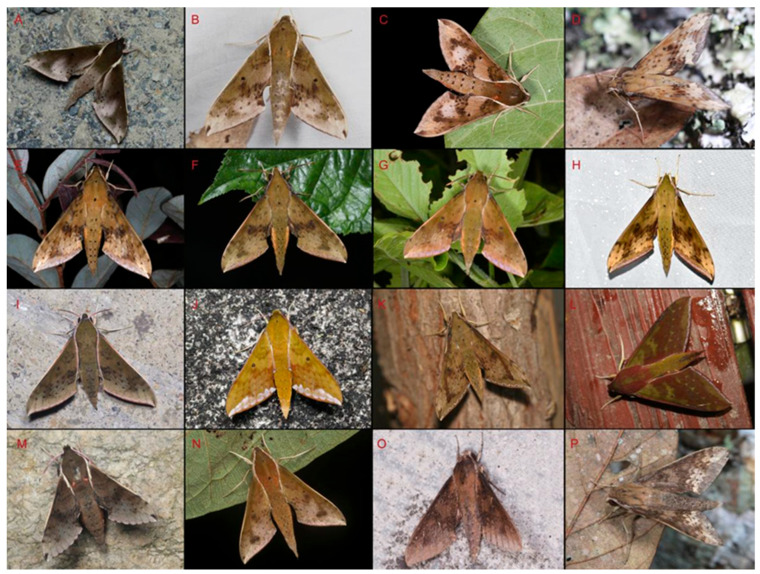
Photos of living individuals of genus *Rhagastis* from China. (**A**). Male *R. albomarginatus*, Lincang, Yunnan, China; (**B**). Female *R. dichroae* stat. nov., Shaoguan, Guangdong, China; (**C**). Male *R. binoculata*, Yilan, Taiwan, China; (**D**). Male *R. aurifera* stat. rev., Xishuangbanna, Yunnan, China; (**E**). Male *R. chinensis* stat. nov., Zunyi, Guizhou, China; (**F**). Male *R. chinensis* stat. nov., Wuxi, Chongqing, China; (**G**). Male *R. formosana* stat. nov., Keelung, Taiwan, China; (**H**). Male *R. jordani* stat. rev., Enshi, Hubei, China; (**I**). Male *R. confusa*, Wuxi, Chongaqing, China; (**J**). Male *R. olivacea*, Shaoguan, Guangdong, China; (**K**). Male *R. lunata*, Yingjiang, Yunnan, China; (**L**). Male *R. gloriosa*, Jingdong, Yunnan, China; (**M**). Male *R. mongoliana*, Mt. Jiugongshan, Hubei, China; (**N**). Male *R. acuta*, Zhaoqing, Guangdong, China; (**O**). Male *R. velata*, Anshun, Guizhou, China; (**P**). Male *R. velata*, Xishuangbanna, Yunnan, China.

Adults of *Rhagastis* (Figure 56) species are nocturnal hawkmoths. According to our observations and collecting experiments, males are easily attracted to light, but females of some species are hard to collect with light traps, such as *R. acuta*, *R. binoculata*, *R. lunata*, *R. aurifera* stat. rev., *R. chinensis* stat. nov., and *R. jordani* stat. rev.

## 4. Discussion

Our morphological comparison is consistent with the molecular evidence. On the basis of this, we elevated *Rhagastis dichroae* Mell, 1922 stat. nov.; *R. everetti* Rothschild & Jordan, 1903 stat. nov.; *R. aurifera* (Butler, 1875) stat. rev.; *R. formosana* Clark, 1925 stat. nov.; *R. chinensis* Mell, 1922 stat. nov.; and *R. jordani* Oberthür, 1904 stat. rev. to full species level.

Our study shows that *R. albomarginatus* and *R. castor* should not be separated into different subspecies. *R. albomarginatus*, *R. binoculata*, *R. dichroae* stat. nov., and *R. everetti* stat. nov. belong to the same species group because they share the same important characteristic: an obvious black discal spot on the hindwing underside. *R. dichroae* stat. nov. whose range includes central and eastern to southeastern China, used to be a subspecies of *R. albomarginatus*, whose distribution covers part of southeastern and southern China, Nepal, Bhutan, India, Vietnam, Laos, and Thailand (Figure 1). The K2P genetic distance (2.78%) (Figure 6) and its different size, pattern, and genitalia structure to *albomarginatus* suggests that it should be a good species (Figure 7, Figure 8, Figure 9, Figure 10, Figure 11 and Figure 12). *R. everetti* stat. nov. is a typical insular species can be recognized easily by it having the largest size and longest forewings in this genus, which is very different from *R. albomarginatus* in its morphological characteristics (Figure 7, Figure 8, Figure 9, Figure 13 and Figure 14) and K2P genetic distance (4.49%) (Figure 6). *R. everetti* is mainly found in Peninsular Malaysia, Sumatra, Java, and Borneo, while the distribution of *R. albomarginatus* does not extend south of central Thailand and southeastern Vietnam, so there is geographical isolation between these two species (Figure 1). *R. aurifera* stat. rev. is related to *R. castor* according the K2P genetic distance (2.48%) (Figure 6), but it is quite different from *R. castor* in its morphological characteristics (Figure 17, Figure 18, Figure 19, Figure 20 and Figure 21) and distribution (Figure 2), being much closer to other species, *e.g.*, *R. chinensis* stat. nov. and *R. confusa*, in appearance (Figure 54), so it is better to treat *R. aurifera* as a good species found in mainland area (China, Nepal, Bhutan, India, Thailand, Laos, Vietnam) while *R. castor* is a typical insular species (Borneo, Sumatra, Java). *R. chinensis* stat. nov. previously had a complex taxonomic status. It was published as *Rhagastis aurifera chinensis* in 1922 with the type locality being “southeastern China” (Figure 22) [16,40], but, subsequently, it has been treated as a synonym of *R. castor jordani* [2]. We checked the *Rhagastis* specimens collected from different localities during 2015 to 2023 alongside the collections of SYSBM and CMNH (four Syntypes) in our morphological and phylogenetic analyses; thus, we believe that *R. chinensis* should be treated as a good species. *R. chinensis* stat. nov. was confused with *R. aurifera* stat. rev. in China for a long time as “*Rhagastis castor aurifera*” and “*Rhagastis acuta aurifera*” [41,42], but we now confirm that they are two separate species according to the K2P genetic distance (5.79%) (Figure 6), and they are synchronous in some areas (Figure 2). Although they are very close in appearance (Figure 54), their male genitalia structure is very different (Figure 21 and Figure 24). The K2P genetic distance also showed that *R. formosana* stat. nov. and *R. chinensis* stat. nov. are very close to each other (2.65%) (Figure 6), and they also share many morphological characteristics like body size, wing patterns, and male genitalic structure. However, they can still be distinguished by their female genitalia (Figure 25 and Figure 28) and geographic distributions. *R. formosana* stat. nov. is currently only known to be found in Taiwan Island of China, while *R. chinensis* stat. nov. covers most areas of central and southern China (Figure 2).

Another complex taxonomic status is that of *R. castor jordani*. According to our field investigation and in the collection of CMNH (Lectotypes and one Paralectotype) (Figure 29), we believe that it deserves the status of a good species only found in central and southwestern China (Shaanxi, Chongqing, Hubei, Sichuan, Guizhou) (Figure 3), as it is totally different from *R. chinensis* and *R. aurifera* not only in morphology (Figure 54) but also in the phylogenetic analysis (Figure 5 and Figure 6). In fact, this taxon should belong to the *olivacea* group rather than the *castor* group because it lacks the lateral orange patch on the abdomen (Figure 54). Our phylogenetic results also suggest its closer relationship to *R. olivacea*, *R. lunata*, *R. gloriosa*, and *R. confusa* rather than to *R. castor*, *R. aurifera* stat. rev., *R. formosana* stat. nov., and *R. chinensis* stat. nov. (Figure 5).

Chu and Wang [41] recorded *Rhagastis mongoliana pallicosta* from Guangdong and Hainan, but according to the description and male genitalia illustration in their book, it appears to be a misidentification of *R. acuta*. They also recorded *Rhagastis miobergi* from the Xizang Autonomous Region in the same book [41]; however, it is a misspelling of *Rhagastis mjobergi*, which is a synonym of *R. rubetra* Rothschild & Jordan, 1907. This species is very similar to *R. acuta* and is found in Thailand, Malaysia, Indonesia, and the Philippines, but it is unlikely to be found in China. Thus, according to the description and male genitalia illustrated in the book, there is no doubt that this *R. miobergi* [sic] is a misidentification of *R. olivacea*. Thirdly, the *Rhagastis acuta aurifera* from Yunnan in the book [41] is a wrong combination of *Rhagastis castor aurifera*; the male genitalia illustration included is actually from a *R. chinensis* stat. nov. The *Rhagastis aurifera* in the book is clearly a misidentification of *Cechenena aegrota* (Butler, 1875), according to the description and illustrations.

**Figure 57:**
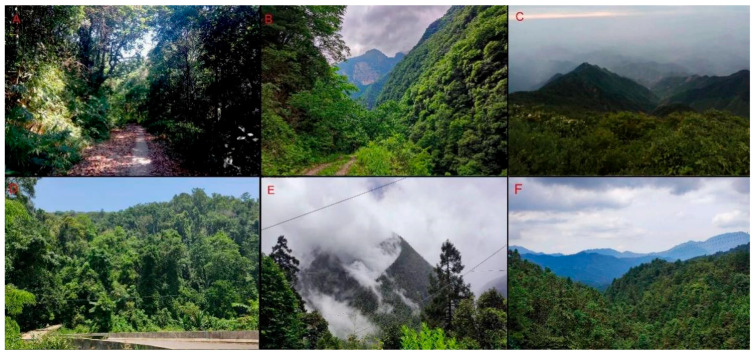
Habitats of genus *Rhagastis* from China. (**A**). Jiangfengling, Hainan, China; (**B**). Wuxi, Chongqing, China; (**C**). Tianmushan, Zhejiang, China; (**D**). Mengla, Xishuangbanna Yunnan, China; (**E**). Zayu, Xizang, China; (**F**). Yuexi, Anhui, China.

China is a country with a mega-biodiversity of hawkmoths (Figure 57), especially in South China, where the physical environment is highly heterogeneous. In recent years, many new taxa and new records of hawkmoths have been described and discovered from this area [42,43,44,45]. Most species in genus *Rhagastis* are forest species; some species can only be found in southwestern China, for example, *R. lunata*, *R. gloriosa*, and *R. aurifera* stat. rev. are only known to be from the Yunnan province and Xizang Autonomous Region in China. According to our study, the genus *Rhagastis* from China includes 14 species now, but further investigation is still needed near the border of China and other countries, especially with regard to the larval records of some species as their life histories are still unknown. Future research must address a better understanding of these species’ divergence, diversity, and distribution pattern when samples from the gap areas become available.

## Figures and Tables

**Table 1:** Sampling information and GenBank accession numbers/BOLD SampleIDs of genus *Rhagastis* samples used in this study. The taxon names follow the current taxonomy mentioned above.

Taxon (Sample Code)	Locality	Collecting Date	GenBank No.	Bold ID
*R. acuta* (JW41)	Jianfengling, Hainan, China	2018-IV-27	PP410222	-
*R. acuta* (JW47)	Ziyuan, Guangxi, China	2023-IV-7	PP410223	-
*R. albomarginatus* (B1)	Jingdong, Yunnan, China	2022-VI-13	PP410199	-
*R. albomarginatus* (B2)	Yingjiang, Yunnan, China	2021-VI-25	PP410200	-
*R. albomarginatus* (JW27)	Motuo, Xizang, China	2022-V-22	PP410201	-
*R. aurifera* stat. rev.	Mengla, Yunnan, China	2012-VII-23	-	ARB00024773
*R. aurifera* stat. rev. (JW9)	Malipo, Yunnan, China	2022-VI-14	PP410217	-
*R. binoculata*	Nantou, Taiwan, China	1992-XII-1	-	BC-Hax2872
*R. binoculata*	Taipei, Taiwan, China	1994-I-1	-	BC-Hax2874
*R. binoculata*	Pingtung, Taiwan, China	2018-VII-2	OQ812083	-
*R. castor* (JW16)	Mt. Halimun, Java, Indonesia	2021-X	PP410212	-
*R. chinensis* stat. nov. (JW1)	Motuo, Xizang, China	2022-IX-10	PP410213	-
*R. chinensis* stat. nov. (JW6)	Yintiaoling, Chongqing, China	2023-VI-26	PP410214	-
*R. chinensis* stat. nov. (JW12)	Shaoguan, Guangdong, China	2019-VI-18	PP410215	-
*R. confusa*	Chiang Mai, Thailand	1998-IX-19	-	BC-Hax2859
*R. confusa*	Jingtang, Sichuan, China	2007-VI-1	-	BC-Mel0936
*R. confusa* (JW36)	Yintiaoling, Chongqing, China	2023-VI-24	PP410218	-
*R. confusa* (JW40)	Jilong, Xizang, China	2020-VII-21	PP410219	-
*R. confusa* (JW41)	Yingjiang, Yunnan, China	2021-VI-25	PP410220	-
*R. dichroae* stat. nov. (JW19)	Leigongshan, Guizhou, China	2023-VIII-16	PP410202	-
*R. dichroae* stat. nov. (JW22)	Huizhou, Guangdong, China	2019-VI-21	PP410203	-
*R. dichroae* stat. nov. (JW28)	Tianmushan, Zhejiang, China	2019-VI-6	PP410204	-
*R. dichroae* stat. nov. (JW31)	Dabieshan, Anhui, China	2023-IX-12	PP410205	-
*R. everetti* stat. nov. (JW24)	Mt. Halimun, Java, Indonesia	2021-X	PP410206	-
*R. everetti* stat. nov. (JW25)	Mt. Halimun, Java, Indonesia	2021-X	PP410207	-
*R. everetti* stat. nov. (JW26)	Mt. Halimun, Java, Indonesia	2021-X	PP410208	-
*R. formosana* stat. nov.	Taitung, Taiwan, China	2004-VIII-1	-	AYK-04-0218
*R. formosana* stat. nov. (JW11)	Pingtung, Taiwan, China	2018-VI-2	PP410216	-
*R. gloriosa*	Mongar, Bhutan	2017-V-29	-	RMNH.INS.1092217
*R. gloriosa*	Yuxi, Yunnan, China	2011-VIII-8	-	ARB00028594
*R. jordani* stat. rev. (JW2)	Libo, Guizhou, China	2017-IV-21	PP410209	-
*R. jordani* stat. rev. (JW7)	Yintiaoling, Chongqing, China	2021-VI-26	PP410210	-
*R. jordani* stat. rev. (JW14)	Baokang, Hubei, China	2022-VII-14	PP410211	-
*R. lunata*	Weibaoshan, Yunnan, China	2000-VII-1	-	BC-Hax2855
*R. lunata*	Xima, Yunnan, China	2001-VI-12	-	BC-Hax2856
*R. lunata*	Weibaoshan, Yunnan, China	2000_VII-1	-	BC-Hax2857
*R. mongoliana*	Lu’an, Anhui, China	2020-VIII-15	OQ589961	-
*R. mongoliana*	Hubei, Luotian, China	2020-VIII-20	OQ589960	-
*R. mongoliana*	Hubei, Yingshan, China	2021-V-2	OQ586404	-
*R. olivacea*	Islamabad, Pakistan	2012-VII-8	-	NIBGE MOT-01958
*R. olivacea*	Trashi Yangste, Bhutan	2017-VI-27	-	RMNH.INS.1092217
*R. olivacea*	Motuo, Xizang, China	2006-VIII-16	-	VAG-267
*R. velata*	Mt. Taunggyi, Myanmar	1993-VIII-12	-	BC-Hax2881
*R. velata*	Chiang Mai, Thailand	2005-VI-23	-	VAG-259
*R. velata* (JW30)	Jinghong, Yunnan, China	2021-VIII-6	PP410221	-

## Data Availability

The data are openly available from GenBank at https://www.ncbi.nlm.nih.gov/genbank/ and BOLD SYSTEMS https://v4.boldsystems.org/ (accessed on 14 January 2024). The list of investigated species and their GenBank accession numbers or BOLD SampleIDs are given in Table 1.

## Appendix A. List of *Rhagastis* Specimens Examined (Different Labels in the Museum Specimens are Separated with a ‘/’)

Names of the institutions in which specimens are deposited are listed after the private collections and are abbreviated as follows: ZHJ—collection of Zhuo-Heng Jiang (Hangzhou, China); CQL—collection of Chang-Qiu Liu (Guilin, China); JSW—collection of Jia-Xin Wang (Hubei, China); HLG—collection of Hao-Lin Gan (Changsha, China); NHMUK—collections of the Natural History Museum (London, United Kingdom); CMNH—collections of the Natural History Museum (Pittsburgh, USA); SM—collections of the Sphingidae museum (Czech Republic); SYSBM—collections of The Museum of Biology, Sun Yat-sen University (Guangzhou, China).

*Rhagastis albomarginatus* (Rothschild, 1894)

2♂♂, Xima (1500 m), Yingjiang, Yunnan, China, 2021-VI-21, Zhuo-Heng Jiang *leg.* [ZHJ]; 3♂♂, Malipo (1310 m), Yunnan, China, 2020-VI-2, Local catcher *leg.* [ZHJ]; ♂♂, Malipo (1450 m), Yunnan, China, 2019-VII-26, Hao-Lin Gan *leg.* [HLG]; ♀, Malipo (1310 m), Yunnan, China, 2020-V-19, Chang-Qiu Liu *leg.* [ZHJ]; 2♂♂, Jingdong (2150 m), Pu’er, Yunnan, China, 2022-VI-9, Zi-Chun Xiong *leg.* [ZHJ]; ♂, Motuo (1200 m), Xizang, China, 2022-V-17, Chang-Qiu Liu *leg.* [ZHJ].

*Rhagastis dichroae* Mell, 1922 stat. nov.

2♂♂, ♀, Tianmushan (763 m), Zhejiang, China, 2016-VII-10, Zhuo-Heng Jiang *leg.* [ZHJ]; 3♂♂, Nankunshan, Huizhou, Guangdong, China, 2022-IV-5, Wei-Cai Xie *leg.* [SYSBM]; 2♂♂, Huizhou (840 m), Guangdong, China, 2019-VI-21, Zhuo-Heng Jiang *leg.* [ZHJ]; 7♂♂, Leigongshan (1200 m), Guizhou, China, 2023-VI-20, Chang-Qiu Liu *leg.* [ZHJ]; 2♂♂, Yuexi (1020 m), Anhui, China, 2023-IX-11, Zhuo-Heng Jiang *leg.* [ZHJ]; ♂, ♀, Yichang (1080 m), Hubei, China, 2023-VIII-27, Qing-Ming Liu *leg.* [ZHJ].

*Rhagastis everetti* Rothschild & Jordan, 1903 stat. nov.

7♂♂, Mt. Halimun, Java, Indonesia, 2021-X, Local catcher *leg.* [ZHJ]; ♂, Mt. Trus Madi, Malaysia, 2018-IV-22, Ye-Jie Lin *leg.* [ZHJ].

*Rhagastis binoculata* Matsumura, 1909

2♂♂, Donghe (560 m), Taitung, Taiwan, China, 2018-VII-2, Wen-Yi Chou *leg.* [ZHJ]; ♂, Taipei, Taiwan, China, 2010-XII-6, Yu-Feng Hsu *leg.* [ZHJ].

*Rhagastis castor* (Walker, 1856)

2♂♂, Mt. Halimun, Java, Indonesia, 2021-IX, Local catcher *leg.* [ZHJ]; ♂, Mt. Trus Madi (1080m), Sabah, Malaysia, 2018-IV-22, Ye-Jie Lin *leg.* [ZHJ]; 3 ♂♂, Kantangan district (800m), S. Kalimantan, Indonesia, 2001-XI, Tomáš Melichar *leg.* [SM].

*Rhagastis aurifera* (Butler, 1875) stat. rev.

♂, Malipo (1370 m), Yunnan, China, 2022-VI-14, Local catcher *leg.* [ZHJ]; ♂, Mengla (950 m), Yunnan, China, 2013-VIII-30, Zhuo-Heng Jiang *leg.* [ZHJ].

*Rhagastis chinensis* Mell, 1922 stat. nov.

♂ SYNTYPE, 7501 S.E. China R. Mell/Clark Collection Acc. 12720/QR Code CMNH-IZ 724561/R. aurifera chinensis, R. Mell/[blue square label] Type [CMNH]; ♂ SYNTYPE, QR Code CMNH-IZ 724563/[blue square label] Type [CMNH]; ♀ SYNTYPE, aurifera chinensis/8162 S.E. China R. Mell/QR Code CMNH-IZ 724565/R. aurifera chinensis ♀ type. Mell/[blue square label] Type [CMNH]; ♀ SYNTYPE, 7502 S.E. China R. Mell/Clark Collection Acc. 12720/QR Code CMNH-IZ 724564/[blue square label] Type [CMNH]; ♀, Lianzhou, Guangdong, China, 1993-IX-11, Xu-Shen Zhong *leg.* [SYSBM]; ♀, Lianzhou, Guangdong, China, 1995-VII-5, Li-Bin Zhu *leg.* [SYSBM]; 2♂♂, Danxiashan, Shaoguan, Guangdong, China, 2012-IV-21, Wei-Cai Xie *leg.* [SYSBM]; ♂, Shaoguan (970 m), Guangdong, China, 2019-VI-18, Zhuo-Heng Jiang *leg.* [ZHJ]; ♂, Motuo (1920 m), Xizang, China, 2023-IX-10, Xin Wang *leg.* [ZHJ]; ♂, Malipo (1370 m), Yunnan, China, 2023-VI-17, Chang-Qiu Liu *leg.* [ZHJ]; ♂, Malipo (1210 m), Yunnan, China, 2021-VI-23, Xiang-Jin Liu *leg.* [ZHJ]; 3♂♂, Maolao (876 m), Libo, Guizhou, China, 2017-IV-21, Zhuo-Heng Jiang *leg.* [ZHJ]; 2♂♂, Yintiaoling (1130 m), Wuxi County, Chongqing, China, 2022-VI-23, Zhuo-Heng Jiang *leg.* [ZHJ].

*Rhagastis formosana* Clark, 1925 stat. nov.

2♂♂, Donghe (560 m), Taitung, Taiwan, China, 2018-VII-2, Wen-Yi Chou *leg.* [ZHJ]; ♂, Alishan (2000 m), Chia-Yi, Taiwan, China, V-16, Schnitzler *leg.* [SM]; ♂, ♀, Kaoshiung, Taiwan, China, V-3, Schnitzler *leg.* [SM]; ♂, Hualien, Taiwan, China, 2008-VII-24, Yu-Feng Hsu *leg.* [JZH]; ♀, Hualien, Taiwan, China, 2006-VI-28, Yu-Feng Hsu *leg.* [JZH].

*Rhagastis jordani* Oberthür, 1904 stat. rev.

♂ LECTOTYPE, Siou-Lou Chasseurs Indigenes Du P. Dejean 1902/9810/QR Code CMNH-IZ 724556/Rhagastis jorani Obths./Rhagastis spec. nov. aurifera n. 795/[pink square label] Type [CMNH]; ♂ PARALECTOTYPE, Siou-Lou Chasseurs Indigenes Du P. Dejean 1902/7340/QR Code CMNH-IZ 724555/Rhagastis jorani Obths. cotype Bull. Soc. ent. France 1904 Etud. Lepid. Comparee, V-pl. LXXI—Figure d type 65/[pink square label] Type [CMNH]; 2♂♂, Yintiaoling (1130 m), Wuxi, Chongqing, China, 2022-VI-23, Zhuo-Heng Jiang *leg.* [ZHJ]; 2♂, Maolan (975 m), Libo, Guizhou, China, 2017-IV-24, Zhuo-Heng Jiang *leg.* [ZHJ]; 2♂, Baokang (1500 m), Xiangyang, Hubei, China, 2022-VII-14, Bin Chou *leg.* [ZHJ].

*Rhagastis confusa* Rothschild & Jordan, 1903

♂, Zhen’an (1650 m), Shaanxi, China, 2014-VII-16, Yu-Fei Li *leg.* [ZHJ]; ♂, Qingchengshan (1120 m), Sichuan, China, 2016-VII-16, Yi Zhang *leg.* [ZHJ]; ♀, Yingjiang (1270 m), Yunnan, China, 2021-VI-25, Zhuo-Heng Jiang *leg.* [ZHJ]; ♂, Jilong (2650 m), Xizang, China, 2020-VII-21, Shuo Qi *leg.* [JZH]; 5♂♂, ♀, Yintiaoling (1130 m), Wuxi, Chongqing, China, 2022-VI-25, Zhuo-Heng Jiang *leg.* [ZHJ]; 3♂♂, Fanjingshan (1600 m), Guizhou, China, 2001-VIII-4, Hong Pang *leg.* [SYSBM]; 2♂♂, ♀, Tacheng (2150 m), Wexi, Yunnan, China, 2023-VIII-14, Local catcher *leg.* [ZHJ].

*Rhagastis velata* (Walker, 1866)

2♂♂, ♀, Yanbian (1840 m), Panzhihua, Sicahun, China, 2019-VIII-5, Zhuo-Heng Jiang *leg.* [ZHJ]; ♂, Jinghong (870 m), Xishuangbanna, Yunnan, China, 2022-VIII-16, Chang-Qiu Liu leg. [ZHJ]; ♀, Yingjiang (1007 m), Yunnan, China, 2023-VII, Wei-Zong Yang *leg.* [ZHJ]; ♂, Anshun (890 m), Guizhou, China, 2019-VI-17, Xin-Yi Zheng *leg.* [ZHJ].

*Rhagastis acuta* (Walker, 1856)

♀, Jianfengling, Ledong, Hainan, China, 1984-VI-1, Li-Zhong Hua leg. [SYSBM]; 3♂♂, Guangzhou, Guangdong, China, 2022-IV-8, Wei-Cai Xie *leg.* [SYSBM]; ♂, Jianfengling, Ledong, Hainan, China, 2017-IV-1, Zhuo-Heng Jiang *leg.* [ZHJ]; ♂, Maolan (975 m), Libo, Guizhou, China, 2017-IV-24, Zhuo-Heng Jiang *leg.* [ZHJ]; 1♂, Ziyuan (1400 m), Guangxi, China, 2017-IV-7, Chang-Qiu Liu *leg.* [ZHJ].

*Rhagastis mongoliana* (Butler, 1876)

2♂♂, Maolan (975 m), Libo, Guizhou, China, 2017-IV-21, Zhuo-Heng Jiang *leg.* [ZHJ]; ♂, Benxi (420 m), Liaoning, China, 2017-V-16, Local Catcher *leg.* [ZHJ]; 5♂♂, Yintiaoling (1130 m), Wuxi, Chongqing, China, 2022-VI-25, Zhuo-Heng Jiang *leg.* [ZHJ]; 2♂♂, Zhaotong (1026 m), Yunnan, China, 2022-VII-12, Peng Wang *leg.* [JZH]; 3♂♂, Yueyang (1020 m), Hunan, China, 2016-IX-10, Zhuo-Heng Jiang *leg.* [JZH]; 5♂♂, Leigongshan (1200 m), Guizhou, China, 2023-VI-20, Chang-Qiu Liu *leg.* [ZHJ]; ♂, Wuzhishan (670 m), Hainan, China, 2017-V-12, Zhuo-Heng Jiang *leg.* [ZHJ]; 2♂♂, Dayaoshan (1120 m), Guangxi, China, 2017-VII-24, Jiang Zhu *leg.* [ZHJ]; 2♂♂, Tiantangzhai, Anhui, China, 2020-VIII-15, Jia-Xin Wang *leg.* [JSW]; 3♂♂, Luotian, Hubei, China, 2021-VIII-20, Jia-Xin Wang *leg.* [JSW]; 4♂♂, Shangcheng, Henan, China, 2020-VIII-17, Jia-Xin Wang *leg.* [JSW]; 3♂♂, ♀, Yingshan, Hubei, China, 2020-VIII-10, Jia-Xin Wang *leg.* [JSW].

*Rhagastis olivacea* (Moore, 1872)

2♂♂, ♀, Motuo (2115 m), Xizang, China, 2018-VII-12, Zhong Peng *leg.* [ZHJ]; ♂, Du-longjiang (1980 m), Yunnan, China, 2023-VI-27, Zhuo-Heng Jiang *leg.* [ZHJ]; ♀, Fuzhou, Jiangxi, China, 2022-VII-30, Local catcher *leg.* [ZHJ]; 2♂♂, Chebaling (1060 m), Guangdong, China, 2016-VII-21, Jiang Zhu *leg.* [ZHJ]; 2♂♂, Dayaoshan (1120 m), Guangxi, China, 2017-VII-24, Jiang Zhu *leg.* [ZHJ]; 6♂♂, Yingjiang (1007 m), Yunnan, China, 2021-VI, Wei-Zong Yang *leg.* [ZHJ].

*Rhagastis lunata* (Rothschild, 1900)

5♂♂, Yingjiang (1007 m), Yunnan, China, 2021-VI, Wei-Zong Yang *leg.* [ZHJ]; 2♂♂, Mt. Gaoligongshan, Yunnan, China, 2016-VI-16, Bin Li *leg.* [ZHJ]; ♂, Motuo (2115 m), Xizang, China, 2018-VII-12, Zhong Peng *leg.* [ZHJ]; ♂, Dulongjiang (1980 m), Yunnan, China, 2023-VI-27, Zhuo-Heng Jiang *leg.* [ZHJ]; ♀, Zayu (1840 m), Xizang, China, 2023-VII-11, Bin Li *leg.* [ZHJ].

*Rhagastis gloriosa* (Butler, 1875)

♂, ♀, Motuo (2115 m), Xizang, China, 2018-VII-12, Zhong Peng *leg.* [ZHJ]; ♂, Jingdong (2150 m), Pu’er, Yunnan, China, 2022-VI-3, Zi-Chun Xiong *leg.* [ZHJ]; ♀, Motuo (2270 m), Xizang, China, 2017-VII-3, Qi-Cheng Yang *leg.* [ZHJ]; 2♂♂, Tacheng (2150 m), Wexi, Yunnan, China, 2023-VIII-12, Local catcher *leg.* [ZHJ].
